# Focal postnatal deletion of *Tsc2* causes epilepsy

**DOI:** 10.3389/fnmol.2025.1686023

**Published:** 2025-11-03

**Authors:** Carlie McCoy, Mary Dusing, Lilian G. Jerow, Grace C. Winstel, Felix Zhan, Jason L. Rogers, Madison Wesley, J. Brian Otten, Steve C. Danzer, Candi L. LaSarge

**Affiliations:** ^1^Division of Neurosurgery, Cincinnati Children’s Hospital Medical Center, Cincinnati, OH, United States; ^2^Department of Anesthesia, Cincinnati Children’s Hospital Medical Center, Cincinnati, OH, United States; ^3^Neuroscience Graduate Program, University of Cincinnati, OH, United States; ^4^Noldus Information Technology Inc., Leesburg, VA, United States; ^5^Department of Anesthesia, University of Cincinnati, Cincinnati, OH, United States

**Keywords:** mTOR, tuberous sclerosis complex, epileptogenesis, cortical development, parvalbumin, somatostatin

## Abstract

**Introduction:**

Tuberous sclerosis complex (TSC) is a genetic disorder caused by mutations in either the *TSC1* or *TSC2* genes. These mutations prevent the TSC1/TSC2 protein complex from forming, resulting in hyperactivation of the mechanistic target of rapamycin (mTOR) cell growth and protein synthesis pathway. Epilepsy is one of the most common neurological symptoms in TSC patients, often associated with focal cortical lesions. However, it is not fully established whether such focal abnormalities are sufficient on their own to generate seizures and associated behavioral deficits. Here, we created a novel mouse model to test the hypothesis that a focal, postnatal deletion of *Tsc2* from cortical neurons is sufficient to induce an epileptogenic network and produce behavioral changes relevant to TSC.

**Methods:**

*Tsc2* was deleted from neurons in a focal area of the frontal cortex in *Tsc2*^fl/fl^ (fTSC2 KO) mice following neonatal bilateral AAV9-CaMKII-Cre-mCherry injections on postnatal day 2. One group of adult fTSC2 KO and *Tsc2*^wt/wt^ (control) mice was implanted with cortical electrodes for combined video-EEG monitoring. A separate group of control and fTSC2 KO mice, injected with a lower viral titer, underwent video recording and behavioral exploration analysis in a novel environment. Tissue was collected for histology.

**Results:**

All adult fTSC2 KO mice implanted with cortical electrodes had seizures, whereas no control mice did. Histological analyses showed that virally infected cells in fTSC2 KO mice had enlarged somas and increased mTOR activation (pS6 expression). These fTSC2 KO mice also had decreased parvalbumin and somatostatin interneuron densities in the surrounding cortex. fTSC2 KO mice displayed increased anxiety-like behaviors, spending significantly less time in the center of the novel environment compared to controls.

**Conclusion:**

A focal, postnatal deletion of *Tsc2* from cortical neurons is sufficient to cause both epilepsy and behavioral deficits in mice. This model recapitulates key phenotypes of TSC, including abnormal cell growth, reduced inhibitory cell density, and increased microglia activation. This fTSC2 KO model is advantageous for delineating the cortical changes that support epilepsy and behavioral deficits in TSC, and for investigating possible targets for therapeutic intervention.

## 1 Introduction

Tuberous sclerosis complex (TSC) is a rare genetic condition associated with abnormal neuron development, intellectual disabilities, and epilepsy. Approximately 1 in 6,000 children born annually are diagnosed with TSC (Kingswood et al., 2017), and most cases are sporadic (Rout and Thomas, 2025). Classified as a “mTORopathy,” TSC is associated with dysfunction of the mechanistic target of rapamycin (mTOR) pathway due to mutations in the *TSC1* (hamartin) or *TSC2* (tuberin) genes. The mTOR pathway is essential for neuronal proliferation, cell growth and development, protein synthesis, and cell survival (for review see Lasarge and Danzer, 2014). Thus, when TSC1 and TSC2 proteins cannot bind to form the negative regulator required for the inactivation of Ras homolog enriched in brain (Rheb), mTOR becomes hyperactivated and causes cell hypertrophy, increased synaptogenesis, and hyperexcitable neural circuits (Fingar and Blenis, 2004; Lasarge and Danzer, 2014; Litwa, 2022). In the central nervous system, the manifestations include seizures, cognitive dysfunction, and autism (Zaroff et al., 2006).

There is no cure for TSC, and epilepsy is one of the most common comorbidities, occurring in 90% of patients (Curatolo et al., 2018). These children have non-cancerous growths in the brain and body, including cortical tubers, subependymal nodules, and subependymal giant cell astrocytomas (Marcotte and Crino, 2006; Rosser et al., 2006; Napolioni et al., 2009). Cortical tubers are commonly the cause of epilepsy; removal of abnormal cells at the lesion site can provide immediate seizure freedom in 70% of patients (Jobst and Cascino, 2015; Liu et al., 2017). However, at ten years post-surgery, about half of patients exhibit seizure recurrence (Specchio et al., 2022). Current pharmacological treatments include Rapalogs; these drugs that dampen mTOR activity have been used to treat TSC with some success (French et al., 2016; Bissler et al., 2017; Franz et al., 2018; Wataya-Kaneda et al., 2018). However, problems with rapalogs include adverse effects, rebound growths, and variable efficacy (for review see Sasongko et al., 2023). Animal models of this disorder are necessary for the development of novel treatment options.

Animal models have been essential for understanding the link between hyperactivated mTOR and epilepsy in patients with TSC. Seizures have been directly linked to mutations in the mTOR pathway (Marsan and Baulac, 2018; Tarkowski et al., 2019), beginning as early as postnatal day 21 in mouse models with *Tsc1* or *Tsc2* deletion and postnatal day 20 with Rheb gain-of-function mutation (Lasarge and Danzer, 2014; Hsieh et al., 2016; Reijnders et al., 2017; Onori et al., 2020). In heterozygous *Tsc1* mice, adult loss of the second allele is sufficient to cause seizures within 9 days, even without overt brain pathology (Abs et al., 2013). Hyperactivation of mTOR, especially in layer II/III neurons of the prefrontal cortex, also caused abnormal cell development with elongated dendrites, enlarged somas, increased axonal projections, and abnormal synapses with enlarged presynaptic terminals (Kwon et al., 2006b; Choi et al., 2008; Chow et al., 2009; Zhou et al., 2009; Tsai et al., 2014).

Many TSC animal models have a severe phenotype with early mortality preventing further investigation (Wilson et al., 2005; Meikle et al., 2007; Way et al., 2009; Zeng et al., 2011; Prabhakar et al., 2013; Koene et al., 2019). mTOR is necessary for regulated cell growth and function, and homozygous knockout of either *Tsc1* or *Tsc2* is embryonic lethal (Onda et al., 1999; Kobayashi et al., 2001). Cre-lox technology has allowed for more specific promoters, developmentally timed gene deletion, or inducible deletion (Kwon et al., 2001, 2006a,b; Marino et al., 2002; Fraser et al., 2004; Yue et al., 2005; Meikle et al., 2007, 2008; Wang et al., 2007; Choi et al., 2008; Zhou et al., 2009; Magri et al., 2011). *In utero* electroporation (IUE) models have been used to target specific developing neurons, through removal of *Tsc1* or *Tsc2* or gain-of-function mutations in Rheb (Feliciano et al., 2011; Tsai et al., 2014; Hsieh et al., 2016; Lin et al., 2016; Lim et al., 2017; Nguyen et al., 2019; Onori et al., 2020; Zhang et al., 2020). However, IUE technology can lead to inconsistent expression due to the diffuse electric field surrounding the targeted region (Yamashiro et al., 2022). It may also affect both neurons and astrocytes derived from the radial glial lineage (Nguyen and Bordey, 2021), and depending on the timing of electroporation, can result in large areas of abnormal neurons (Lim et al., 2015; Hsieh et al., 2016). Such factors can make it challenging to attribute experimental outcomes specifically to the targeted genetic change, rather than to confounding effects on multiple cell types or broader developmental abnormalities.

To achieve more precise spatial and temporal control, we employed a viral-mediated approach to test the hypothesis that deletion of *Tsc2* from a localized region of cortical neurons at postnatal day 2 was sufficient to cause the formation of an epileptogenic network. Indeed, this manipulation led to mTOR upregulation, hypertrophic neurons, epilepsy, and anxiety-like behavior. Moreover, most focal *Tsc2* knockout (fTSC2 KO) mice survived until adulthood, when EEG monitoring (68% survival) and behavioral testing (100% survival) could occur. This fTSC2 KO model is advantageous for investigating TSC-associated epileptogenesis and accompanying behavioral deficits.

## 2 Materials and methods

### 2.1 Mice

All animal studies were in accordance with the NIH *Guide for the Care and Use of Laboratory Animals (National Research Council (US) Committee for the Update of the Guide for the Care and Use of Laboratory Animals, 2011)* and in agreement with CCHMC Institutional Animal Care and Use Committee (IACUC) guidelines and approval. *Tsc2^fl/wt^* mice (RRID:IMSR_JAX STRAIN#027458; Tsc2 < tm1.1Mjg > /J) containing loxP sites flanking exons 2, 3, and 4 of the *Tsc2* gene (Hernandez et al., 2007) were outbred for at least 7 generations with C57BL/6 mice. EEG study animals were generated by crossing *Tsc2^fl/wt^* mice to produce *Tsc2^fl/fl^* (*Tsc2* “floxed”) and littermate *Tsc2^wt/wt^* (*Tsc2* “wildtype”) mice. Behavioral study mice were made by breeding *Tsc2^fl/wt^*, TdTomato*^fl/fl^* (RRID:IMSR_JAX:007914; B6.Cg-*Gt(ROSA)26Sor*^*tm*14(*CAG–tdTomato*)*Hze*^/J) mice with *Tsc2^fl/wt^* mice to produce *Tsc2* wildtype and floxed mice with one allele of *TdTomato* (TdTom +). Previous *Tsc2*^±^ mouse models did not show a seizure phenotype (Kobayashi et al., 1999, 2001; Onda et al., 1999; Ehninger et al., 2008); in line with our hypothesis, *Tsc2^fl/wt^* mice were not tested. All mice were maintained on a C57BL/6 background, and littermate controls were used when possible. All data collection and analyses were conducted with investigators blind to animal genotype and treatment.

### 2.2 Postnatal day two viral injections

**FIGURE 1:**
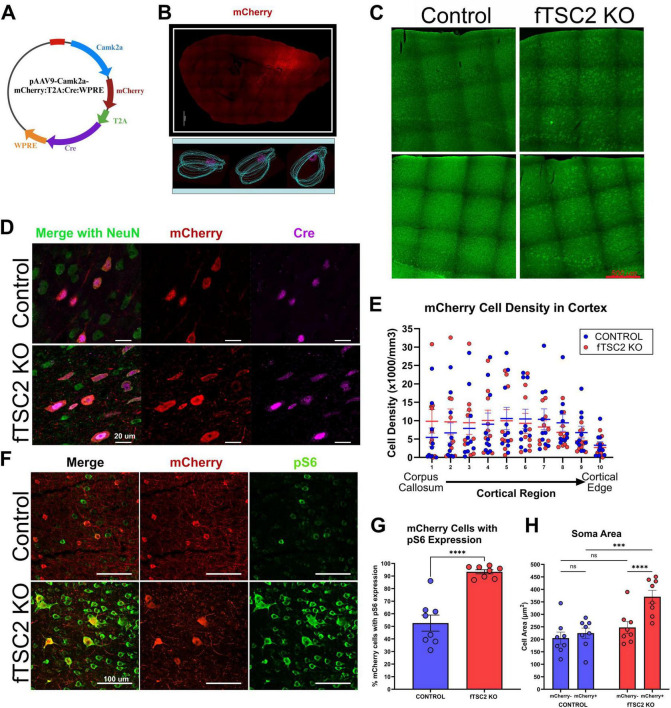
mCherry expression indicates a focal area of *Tsc2* KO cells. **(A)** CamkIIa-mCherry-T2A-Cre was packaged into an AAV9 for injection into the cortex of *Tsc2^fl/fl^* and *Tsc2*^wt wt^ mice. Viral map created using BioRender. **(B)** Tile scan image showing the location of mCherry expression cells in a *Tsc2*^fl/fl^ mouse injected with CamkIIa-mCherry-T2A-Cre at postnatal day 2. Images of the right hemisphere were traced using contours in Neurolucida (MBF Bioscience) with the pink lines highlighting mCherry expression. Scale bars = 1,000 μm. **(C)** Control and focal *Tsc2* knockout (fTSC2 KO) tissue was stained with NeuroTrace. Images demonstrate a normal cortical lamination in fTSC2 KO mice. **(D)** The number of mCherry+ cells in cortex of control and fTSC2 KO tissue was comparable; however, there was a regional difference in both groups *p* < 0.001, with fewer mCherry + cells in region 10 near the cortical edge when compared to regions 1–8 (*p* ≤ 0.01) and in region 9 compared to regions 5 and 6 (*p* ≤ 0.028). **(E)** Tissue from control and fTSC2 KO mice was immunostained for Cre and mCherry; coincidence of the mCherry in cells that express Cre suggested mCherry could be used to identify TSC2 KO cells. Scale Bar = 20 μm. **(F)** Tissue from control and fTSC2 KO mice were immunostained for mCherry and pS6. Scale bar = 100 μm. **(G)** pS6 was highly expressed in fTSC2 KO tissue compared to controls, with an average of over 90% of mCherry+ cells in the fTSC2 expressing pS6 compared to 53% of control mCherry + expressing pS6. **(H)** The soma area of pS6 expressing mCherry– and mCherry+ neurons in the lesion area were measured in control and fTSC2 KO tissue; mCherry+ cells in the *Tsc2* floxed mice were significantly larger than mCherry– cells in the same tissue and larger than mCherry+ cells in control tissue. ***, *p* < 0.001; *⁣*⁣**, *p* < 0.0001.

AAV9-CaMKIIa-mCherry-T2A-Cre (Figure 1A; Vector builder, Chicago, IL) was injected bilaterally into focal (f) areas of the cortex of *Tsc2* wildtype (control; males, *n* = 6; females, *n* = 4) and *Tsc2* floxed (fTSC2 KO; males, *n* = 15; females, *n* = 10) pups at postnatal day 2 for the excision of the *loxP*-flanked *Tsc2* gene and expression of mCherry. The stereotaxic frame was maintained at 2–4 degrees Celsius using chilled 100% EtOH and dry ice in the frame well [modified from Kim et al. (2014)]. Pups were placed on a Kimwipe covered, chilled (0°) aluminum block until unresponsive. Pups were head fixed, and a Neuros Syringe (0.5 μl, 32G, beveled; Cat# 65457-02) was inserted through the skull into the cortex (A/P: −0.5 mm; M/L: ± 0.5 mm; D/V −0.35 mm and A/P: −1.0 mm; M/L: ± 0.65 mm; D/V −0.35 mm) for 4 injections (50 nl at 100 nl/min; virus of 3.55 × 10^9^ gc/μl with 0.05% Trypan Blue) using an automated syringe pump. Pups were then warmed before they returned to the mother. Half of the litter was removed for injections, then swapped. Pups were tailed and genotyped on days 10–14 and weaned on day 28. Of 22 fTSC2 KO mice generated for EEG recording, 7 (32%) died before implant, between 7 and 11.86 weeks of age (9.633 ± 0.700 weeks; five males and two females). Ten fTSC2 KO mice (six males and four females chosen randomly) received EEG implants, and 5 fTSC2 KO mice (two males and three females) weren’t used for the study. There was no mortality among the 10 control mice assigned for implant.

### 2.3 EEG analysis

Control (males, *n* = 6; females, *n* = 4) and fTSC2 KO mice (males, *n* = 6; females, *n* = 4) balanced for sex and age were implanted with cortical surface electrodes connected to wireless EEG transmitters (1 channel, TA11ETA-F10, Data Sciences International, St. Paul, MN). EEG recorded mice ranged in age from 8.43 to 18.00 weeks at implantation (controls: 10.19 ± 1.00; fTSC2 KO: 10.73 ± 0.96 weeks; t_(18)_ = 0.390, *p* = 0.702). Cortical electrodes were placed under the skull above the dura, bilaterally (two electrodes, ± 1.5 mm lateral, 0.5 mm posterior to bregma). Mice were provided with Carprofen (40 mg/kg) for pain post-surgery and 24 h later, and antibacterial ointment was applied to the wound for 3 days. Video-EEG data was collected 24 h per day, 7 days per week, and reviewed using NeuroScore software (Version 2.1.0, Data Sciences International) to identify seizures. EEG events scored as seizures were characterized by the sudden onset of high amplitude (> 2x background) activity, signal progression (a change in amplitude and frequency over the course of the event) and a duration greater than 10 s (LaSarge et al., 2021). Mice were recorded from for at least 7 days (controls: 11.20 ± 0.854 days; fTSC2 KO: 8.00 ± 0.300 days); any bias from controls recording longer would have been toward seizure detection. At the end of recording, mice were overdosed with pentobarbital (100 mg/kg) and perfused with phosphate buffered saline (PBS) + 1 U/ml heparin, followed by 2.5% paraformaldehyde with 4% sucrose in PBS (pH 7.4). Brains were post-fixed overnight, cryoprotected, frozen, and stored at −80 °C. Frozen brains were sectioned in the sagittal plane on a cryostat at 30 μm. Four sections, spaced 540 μm apart, were mounted to each gelatin-coated slide and stored at −80 ° until use.

### 2.4 Immunohistochemistry and histological analysis

#### 2.4.1 Immunolabeling

Immunolabeling was performed on slide mounted tissue from EEG recorded control and fTSC2 KO mice. For visualization of Cre and mCherry co-expression in cells, as well as inhibitory cell counts, tissue was permeabilized overnight in 3% Triton-100 and 0.75% glycine in PBS, washed, and blocked with 1.5% Triton-100, 0.75% glycine, and 5% normal goat serum (NGS) or normal donkey serum in PBS, respectively. Slides to be assessed for pS6, as well as those assessed for inflammatory markers glial fibrillary acidic protein (GFAP) and ionized calcium-binding adaptor molecule 1 (IBA1), were treated overnight with 0.5% Igepal, 3% Tween 20, and 0.75% glycine in PBS, washed, and blocked in 5% NGS, 0.5% Igepal, and 0.75% glycine in PBS.

Following block, all tissue was incubated overnight with antibodies (see Table 1); a 4-h incubation with corresponding AlexaFluor secondary antibodies (Table 1) was followed by cover-slipping with ProLong Glass containing NucBlue (Thermo Fisher Scientific Cat #P36981).

**TABLE 1:** Primary and secondary antibodies used for histological analyses.

Primary antibodies	Secondary antibodies/stains
Mouse anti-NeuN (1:200 Millipore Cat#MAB377, RRID:AB_2298772)	AlexaFluor 488 goat anti-mouse (1:750; Thermo Fisher Scientific Cat# A28175, RRID:AB_2536161)
Chicken anti-mCherry (1:750 Sigma Cat#ab356481, RRID:AB_2861426)	AlexaFluor 568 goat anti-chicken (1:750; Thermo Fisher Scientific Cat# A-11041, RRID:AB_2534098)
Rabbit anti-CRE (1:750 Cell Signaling Cat#15036, RRID:AB_2798694)	AlexaFluor 647-plus goat anti-rabbit (1:750; Thermo Fisher Scientific Cat# A-21244, RRID:AB_2535812)
Rabbit anti-phospho-240/244-S6 (pS6 antibody, 1:500 dilution, Cat# 9468 RRID:AB_2716873)	Alexa Fluor 647 goat anti-rabbit (Thermo Fisher Scientific Cat# A32733TR, RRID:AB_2866492)
Chicken anti-mCherry (1:750 Sigma Cat#ab356481, RRID:AB_2861426)	AlexaFluor 568 goat anti-chicken (1:750; Thermo Fisher Scientific Cat# A-11041, RRID:AB_2534098)
	NeuroTrace 500/525 (1:300, Thermo Fisher Scientific Cat# N21480)
Rabbit anti-SST (1:500; Thermo Fisher Scientific Cat# PA5-82678, RRID:AB_2789834)	Alexa Fluor 488 donkey anti-rabbit (1:750, Thermo Fisher Scientific Cat# A32790, RRID:AB_2762833)
Chicken anti-mCherry (1:750, Sigma Cat# ab356481, RRID:AB_2861426)	Alexa Fluor 568 donkey anti-chicken (1:750, Thermo Fisher Scientific Cat# A78950, RRID:AB_2921072)
Guinea pig anti-PV (1:1000; Synaptic Systems Cat# 195 004 RRID:AB_2156476)	Alexa Fluor 647 donkey anti-guinea pig (1:750, (Jackson Labs Cat# 706-605-148, RRID:AB_2340476)
Chicken anti-GFAP (1:500 Millipore Cat#AB5541, RRID:AB_177521)	Alexa Fluor 488 goat anti-chicken (Thermo Fisher Scientific Cat# A32931, RRID:AB_2762843)
Rabbit anti-IBA1 (1:500 Synaptic Systems Cat#234 008, RRID:AB_2891296)	Alexa Fluor 647-plus goat anti-rabbit (Thermo Fisher Scientific Cat# A-21244, RRID:AB_2535812)

Primary and secondary antibodies used during immunohistochemistry experiments at the dilutions indicated.

#### 2.4.2 Imaging and analysis

All imaging was conducted at the Bio-Imaging and Analysis Facility at Cincinnati Children’s Hospital Medical Center (RRID:SCR_022628) using a Nikon Eclipse Ni-E upright microscope with a 4X (NA 0.20) and 10X (NA 0.45) air objectives or a Nikon A1R confocal microscope equipped with 10X air (NA 0.45), 20X water-immersion (NA 0.95, resolution 0.62 μm/px), and 40X water-immersion (NA 1.15, resolution 0.31 μm/px) objectives (Nikon Instruments Inc.; RRID:SCR_020317). For 3D lesion reconstruction, at least three slides per animal, with at least three sections of tissue (9 ≤ sections total), were imaged at 4 × magnification spanning from lateral ± 0.12 to 3.00 mm. Cortex size and lesion area were traced by hand, to include cell bodies of mCherry + cells, using NIS-Elements AR imaging software (version 5.24.03; NIS-Elements, RRID:SCR_014329). The cortex and lesion were reconstructed using section number and thickness, and volumes were estimated.

For confocal images, scanning began 3 μm below the tissue surface to exclude damaged areas. Cre immunostaining was imaged using a 2 μm step for 4 μm through the z-axis. pS6, mCherry, NeuroTrace, SST, and PV immunostaining images were acquired using a 1 μm step for 18–23 μm through the z-axis; large images extended out at least 800 μm from the center of the viral-labeled cells. GFAP and IBA1 immunostaining images were acquired using a 0.5 μm step for 6 μm through the z-axis. A single optical image of NeuroTrace was also collected at the site of the lesion to assess cortical layering. Large images included the area of the lesion and at least 500 μm of tissue from the lesion.

For analysis, image stacks were loaded into Nikon NIS-Elements, except for mCherry and inhibitory cell counts that were completed using Neurolucida 360 (Version 2023.1.1, MBF Bioscience, VT; RRID:SCR_001775). All analyses were performed by investigators blinded to genotypes. Cellular hypertrophy driven by the hyperactivation of mTOR is a characteristic of TSC-associated lesions. To calculate cell size, a 100 μm square grid was placed over the image and 20 random squares in the lesion were selected between cortical layers II-IV; the circumference of a randomly selected neuron expressing pS6 and mCherry and a neuron with only pS6 expression (mCherry negative) were traced using the area measurement tool. pS6, mCherry, SST, and PV cell markers were counted in each section of cortex, spanning from the outer cortical edge to the corpus collosum. Absolute cell counts were performed; cells with intensity levels at twice the background were considered positive for the marker. Contours were used to distinguish the corpus callosum from the edge of the cortex, and cortex was divided into 10 equal areas between the contours to account for any changes in the cortical thickness or organization of cortical layers, similar to previously described methods (Zhong et al., 2021). Cell count and location data were exported into Microsoft Excel (Microsoft 365 version 2401, Microsoft Corp., RRID:SCR_016137), where cell densities were calculated for each individual animal. For each GFAP and IBA1 image, the percentage of the image with signal from cell labeling was calculated for each animal.

### 2.5 Behavioral exploration analysis

Behavioral analyses were conducted on a separate cohort of mice from EEG studies at a comparable age [range (11.71–16.43 weeks); mean control: 14.80 ± 0.55; mean fTSC2 KO: 14.75 ± 0.25]. Due to the severity of seizures in the cohort of mice that underwent EEG recording, mice for behavioral studies were injected with a reduced viral titer. TdTom + *Tsc2* wildtype (control males, *n* = 6; females, *n* = 2) and TdTom + *Tsc2* floxed (fTSC2 KO males, *n* = 3; females, *n* = 6) pups were injected with a combined 1:10 dilution of AAV9-CaMKIIa-mCherry-T2A-Cre (3.55 × 10^8^ gc/μl with 0.05% Trypan Blue) and AAV9-CaMKIIa-eGFP (3.55 × 10^9^ gc/μl with 0.05% Trypan Blue; Addgene cat# 50469-AAV9) in the same manner as described for EEG recorded mice. This fTSC2 KO cohort had no mortality, and 2 mice had behavioral seizures in the presence of the investigator.

Control and fTSC2 KO mice were placed alone in a new circular cage (10 in diameter and 8 in wall height) with a top-mounted camera in a new room to test their exploration of a novel environment. Mice were tested in a room with mice of the same sex, in randomized groups of four that contained both control and fTSC2 KO mice. Animals were tracked for the first 30 min in this cage using EthoVision XT (v17.5, Noldus Information Technology, Leesberg, VA, RRID:SCR_000441). The center zone was defined as the middle 4 inches of the field. The distance moved and center zone duration for each mouse was split into 5-min time bins and compared over time. Mice were perfused and tissue collected after all behavioral testing was complete (control: 16.64 ± 0.03 weeks; KO: 16.83 ± 0.04 weeks). Tissue was processed, cut, and analyzed for lesion size in the same manner as the tissue from the EEG analysis.

### 2.6 Statistics

Sex differences were analyzed for each measure using either a *t*-test or a two-way ANOVA (with genotype), although none were significant. Since no sex differences were detected, males and females were binned for analyses. Due to low n’s, however, the lack of a sex effect should be interpreted cautiously. All results are presented as mean ± SEM or medians (range). The number of animals are reported as “n,” unless otherwise noted. Statistical tests were performed using Sigma Plot software (version 14.0, Systat Software, Inc., RRID:SCR_003210) or Prism (version 10.1.2, GraphPad Software, LLC., RRID:SCR_002798). Graphs were made in Prism. Parametric tests were used for data that met assumptions of normality (Shapiro-Wilk test) and equal variance (Brown-Forsythe test), and non-parametric equivalents were used for data that did not meet these assumptions. Specific tests were used as noted in the results. Results are considered as significantly different if *p* < 0.05, unless otherwise noted.

### 2.7 Figure preparation

Microscopy images are either single confocal optical sections or confocal maximum projections. Some images were adjusted using Nikon NIS-Elements (NIS-Elements, RRID:SCR_014329) with a median filter (radius = 3) to reduce background artifacts. Brightness and contrast of digital images were adjusted to optimize cellular detail. Identical adjustments were made to all images meant for comparison. Figures were prepared using Adobe Photoshop (version 25.5.1, Adobe Photoshop, RRID:SCR_014199). Figures created using BioRender are specified in the legend.

## 3 Results

### 3.1 Viral deletion of *Tsc2* in cortical cells

EEG recorded *Tsc2* wildtype (control) and *Tsc2* floxed (fTSC2 KO) mice injected on postnatal day 2 with an AAV9-CamKII-mCherry-T2A-Cre virus (Figure 1A) expressed mCherry in cortical neurons. mCherry expressing (+) neurons were found in a focal area of the frontal cortex, spanning from layer VI at the edge of the corpus callosum to layer I at the cortical edge (Figures 1B, E). A comparison of the cortical volume between the control mice and fTSC2 KO mice revealed no difference between groups (control, *n* = 10: 24.251 ± 0.744 mm^3^ mean ± SEM); fTSC2 KO, *n* = 10: 28.384 ± 2.019 mm^3^; *t*-test [t(18) = 1.920, *p* = 0.071]. Next, the volume of the cortex with mCherry expressing cells was calculated and normalized to the volume of the cortex. The percentage of the cortex with infected cells (lesion size) was also comparable in control and fTSC2 KO mice control: 32.623 ± 3.498; fTSC2 KO: 39.165 ± 1.810; *t*-test [t(18) = 1.661, *p* = 0.114]. Lastly, fTSC2 KO mice had no apparent changes in cortical lamination when compared to controls, including a distinct layer I at the cortical edge (Figure 1C).

The distribution of mCherry + cells across cortical layers was examined to determine which regions contained virally infected cells. This analysis aimed to characterize the spatial pattern of infection and assess whether specific cortical layers were preferentially targeted by the viral manipulation. To account for potential structural changes in cortical layering due to *Tsc2* deletion, the cortex was divided into 10 equal regions, similar to Zhong et al. (2021). Within each region, the number of mCherry^+^ cells was counted and normalized by the region’s volume to calculate cell density. The overall density of mCherry^+^ cells was comparable between control (*n* = 10) and fTSC2 KO (*n* = 9) mice Figure 1E; Two-way ANOVA [F_geno_(1,17) = 0.0002, *p* = 0.988; F_region_(9,153) = 6.309, *p* < 0.001], indicating that the viral infection produced a similar lesion in both groups. However, cell distribution varied significantly across cortical regions. Both groups had fewer mCherry + cells near the cortical edge (region 10) compared to deeper regions of the cortex (region 1–8; *p* ≤ 0.01, Tukey correction for multiple comparisons), and fewer cells in superficial regions (region 9) when compared to middle regions (regions 5 and 6; *p* ≤ 0.028). Estimates of mCherry + cells per lesion were calculated by multiplying the estimated lesion volume by the estimated mCherry + cell density. These calculations revealed no significant difference in total mCherry + cells between control and fTSC2 KO mice control: 75,188 ± 28,587; fTSC2 KO: 98,173 ± 31,606; *t*-test [t(17) = 0.5408, *p* = 0.882].

Cre expression, pS6 expression, and cell size were used to indicate that *Tsc2* activity was disrupted in mCherry + cells in the fTSC2 KO mice. The viral plasmid contained genes for both mCherry and Cre; however, the T2A would allow them to be expressed separately (Figure 1A). mCherry expression was visually compared to Cre expression in cortical neurons at the injection site in control and fTSC2 KO tissue using immunohistochemistry; indeed, mCherry expression overlapped with Cre expression, suggesting that mCherry could be used to identify TSC2 KO cells in the *Tsc2* floxed mice (Figure 1D). Next, pS6 expression, the product and readout used to assess mTOR activation (Ma and Blenis, 2009), was compared in control and fTSC2 KO tissue. Early data collected from this model showed mCherry + cells in the fTSC2 KO animal were negative for TSC2 expression (Dusing et al., 2023). Since only a part of the *Tsc2* gene was deleted, pS6 expression was used here to show a loss of functionality, which would present as an upregulation of mTOR activity. As seen in Figure 1F, pS6 was highly expressed in fTSC2 KO tissue compared to the control. Specifically, 93.36 ± 1.643% of mCherry + cells expressed pS6 in the fTSC2 KO tissue (*n* = 8), while only 52.60 ± 6.390% of neurons in the mCherry + cells control tissue (*n* = 8) expressed pS6 Figure 1G; *t*-test [t(14) = 6.177, *p* < 0.001].

mTOR hyperactivation in neurons can lead to increased soma sizes, and the impact of *Tsc2* deletion was assessed. Since almost all mCherry cells in the fTSC2 KO tissue expressed pS6, and mTOR is associated with growth and protein synthesis, only neurons expressing pS6 in all conditions were measured to prevent bias. Unfortunately, due to the small population of mCherry + cells lacking pS6 activation in the *Tsc2* floxed mice, we were unable to reliably quantify the soma size of mCherry + /pS6 negative cells. As demonstrated in Figure 1F, the pS6 and mCherry labeling were both localized to the cytoplasm and spatially overlapped within the same cells, enabling accurate soma size measurements in mCherry+ and mCherry− cells. The soma area of pS6 positive neurons with and without mCherry expression were measured and compared between the control (*n* = 8) and fTSC2 KO (*n* = 8) tissue Figure 1H; 2way RM ANOVA [F-inx_mCherry__x__geno_(1,14) = 15.99, *p* = 0.0013; F_geno_(1,14) = 9.512, *p* = 0.008; F_mCherry_(1,14) = 30.58, *p* < 0.0001]. As shown by the interaction, mCherry + cells in the fTSC2 KO mice were significantly larger than mCherry + cells from control mice [control_mCherry+_: 224.52 ± 20.37; fTSC2 KO_mCherry+_: 370.64 ± 25.61; Fisher’s LSD_(mCherry+:_ _control__v__fTSC2_ _KO)_: *p* = 0.0001], but not mCherry negative cells [control_mCherry_−: 204.71 ± 24.03; fTSC2 KO_mCherry_−: 247.41 ± 23.66; Fisher’s LSD_(mCherry–:_ _control__v__fTSC2_ _KO)_: *p* = 0.209]. Moreover, mCherry + cells in the *Tsc2* floxed mice were significantly larger than mCherry negative cells in the same tissue [Fisher’s LSD_(fTSC2_ _KO:_ _mCherry+__v__mCherry–)_: *p* < 0.0001]. In the control tissue, the soma size of neurons with and without mCherry were comparable [Fisher’s LSD_(Control:_ _mCherry+__v__mCherry)_: *p* = 0.297]. Altogether, mCherry + cells in *Tsc2* floxed tissue that expressed Cre, largely expressed pS6, and had increased soma area were TSC2 KO cells.

### 3.2 Focal deletion of *Tsc2* from cortical neurons causes seizures

**FIGURE 2:**
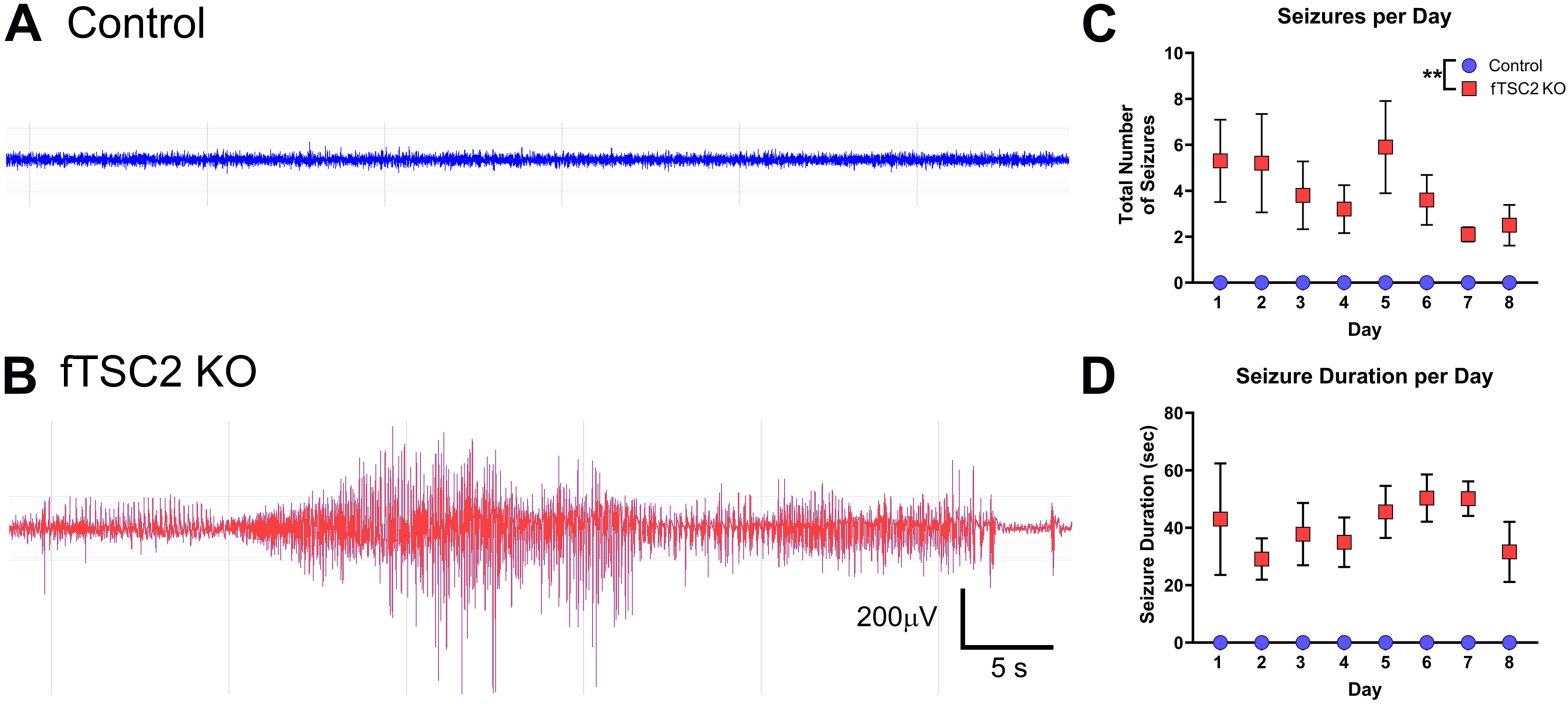
Focal *Tsc2* knockout (fTSC2 KO) mice have seizures. **(A)** Control and **(B)** fTSC2 KO mice were implanted with cortical EEG electrodes and monitored 24/7 for 7–12 days. Seizures were quantified using NeuroScore software, characterized by the sudden onset of high amplitude (> 2X background) synchronized activity, signal progression (a change in amplitude or frequency over the course of the event), and a duration greater than ten seconds. All fTSC2 KO mice had consistent class 5 seizures. **(C)** fTSC2 KO mice consistently had an average of 5.26 ± 0.69 (mean ± SEM) seizures per day. No control mice had seizures. **(D)** fTSC2 KO mice had seizures that lasted on average 42.17 ± 2.12 s each and were consistent during the recording period. **, *p* < 0.01.

Control and fTSC2 KO mice were implanted with EEG electrodes for 24/7 (hours per day/days per week) seizure detection and video monitoring. The number and duration of EEG events classified as seizures were recorded for each animal for at least 7 days. No control animals had any seizures (characteristic control EEG shown in Figure 2A). However, every fTSC2 KO mouse, including mice implanted at 8 weeks of age, had seizures (average 3.950 ± 0.490 seizures per day; representative EEG seizure, Figure 2B) accompanied by behavioral symptoms (i.e., myoclonic jerking, forelimb clonus, rearing, and falling). Analysis of the seizures per day over the recording period showed only a significant difference between control (*n* = 10) and fTSC2 KO mice (*n* = 10); seizures did not vary by day Figure 2C; Two-way ANOVA [F_geno_(1,18) = 37.46, *p* < 0.0001; F_day_(7,119) = 0.878, *p* = 0.526]. Seizure duration was also consistent over all recording days Figure 2D; Two-way ANOVA [F_geno_(1,18) = 106.500, *p* < 0.0001; F_day_(7,119) = 0.569, *p* = 0.780]. Seizures also did not correlate with the age of the fTSC2 KO mouse [Pearson r correlation: (r_age_ = 0.124, *p* = 0.733)]. Altogether, fTSC2 KO mice had a consistent seizure phenotype over the recording period.

### 3.3 Loss of PV and SST inhibitory cells in the cortex of fTSC2 KO mice

**FIGURE 3:**
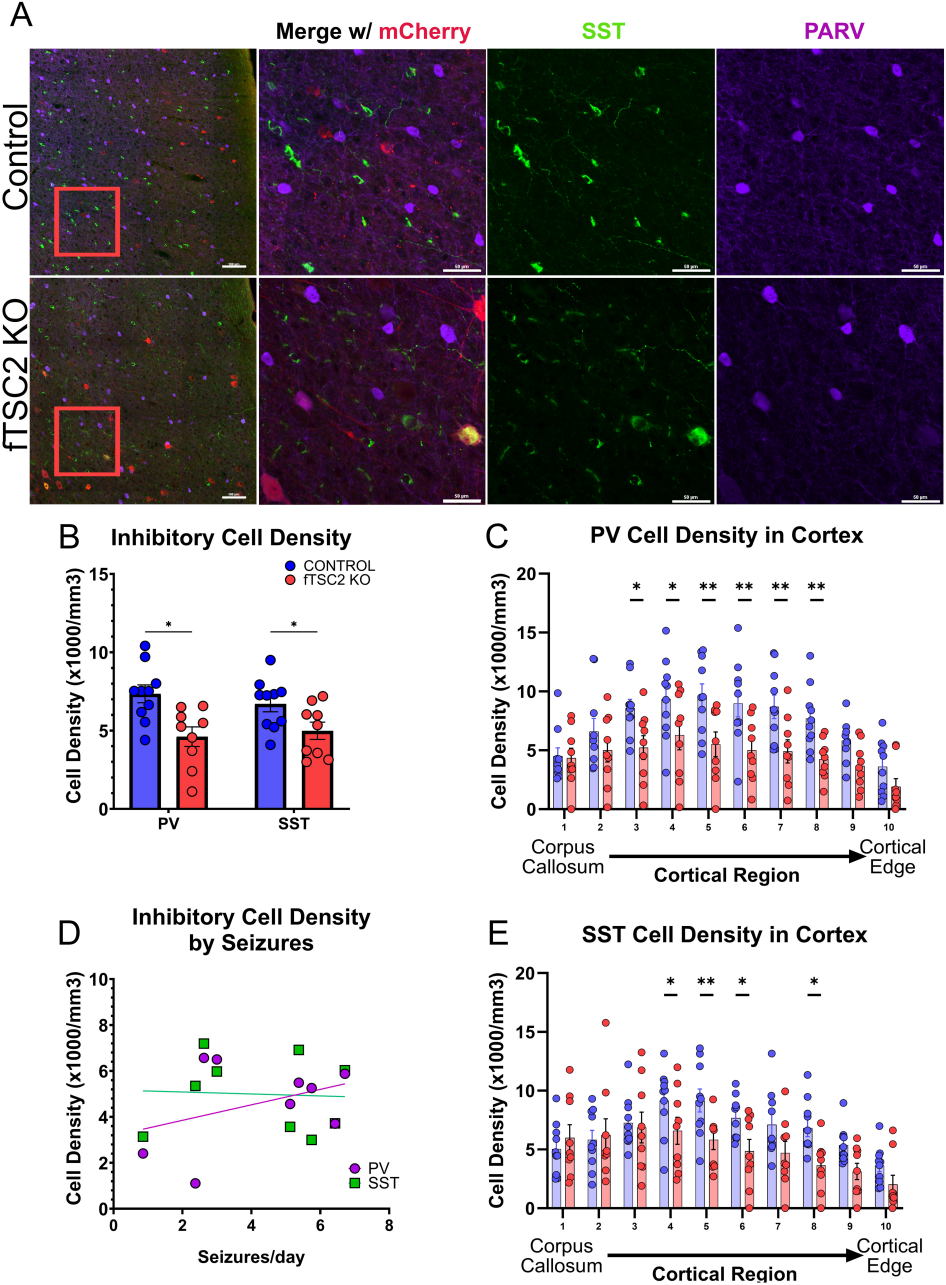
Focal *Tsc2* knockout (fTSC2 KO) mice have decreased somatostatin and parvalbumin cells in the cortex. **(A)** Tissue from control and fTSC2 KO mice were labeled with antibodies for somatostatin (SST), mCherry, and parvalbumin (PV). Scale bars = 100 (left, 10X) and 50 μm (40X). **(B)** Parvalbumin (PV) and Somatostatin (SST) interneuron densities were decreased in fTSC2 KO mice compared to controls (*p* < 0.05). **(C)** PV cell densities were decreased in the fTSC2 KO mice compared to controls, particularly in regions 3–8 (*p* < 0.05 or *p* < 0.01, as indicated). **(D)** Inhibitory cell densities were not correlated with seizures. **(E)** SST cell densities were decreased in the fTSC2 KO mice compared to controls, particularly in regions 4–6, and 8 (*p* < 0.05 or *p* < 0.01, as indicated). *, *p* < 0.05; **, *p* < 0.01.

Tuberous sclerosis complex is frequently associated with dysfunctional inhibition (Valencia et al., 2006; Delia et al., 2012; Bateup et al., 2013b; Zhong et al., 2021; Aronica et al., 2023; Scheper et al., 2024), which can further disrupt the excitatory-inhibitory balance and contribute to a more severe seizure phenotype. Tissue from control (*n* = 10) and fTSC2 KO (*n* = 9) mice was immunostained for parvalbumin (PV) and somatostatin (SST) to determine whether the fTSC2 KO mice had disrupted inhibitory circuit architecture that could contribute to an altered excitatory-inhibitory balance. PV and SST inhibitory cells, as shown in Figure 3A, were detected throughout the cortical layers, as typically seen in mice (Wall et al., 2016). Analyses across combined cortical layers revealed a reduction in interneuron cell density in fTSC2 KO tissue compared to controls (Figure 3B). Specifically, PV + control: 7347 ± 570; fTSC2 KO: 4609 ± 625; *t*-test [t_PV_(17) = 3.242, *p* = 0.005]) and SST (control: 6706 ± 503; fTSC2 KO: 4988 ± 550; *t*-test [t_SST_(17) = 2.309, *p* = 0.034] interneurons were decreased in the lesion and the surrounding 500 μm region. These findings suggested a disruption in the local inhibitory network in the fTSC2 KO mice.

To assess whether interneurons in specific cortical regions exhibited heightened vulnerability in this model, the cortex was subdivided into 10 equal regions spanning the cortical layers to remove bias from potential laminar organization (Zhong et al., 2021). Regional analysis revealed differential changes in both PV and SST interneuron populations in fTSC2 KO tissue, suggesting that inhibitory circuits may be impacted differently across cortical depths. While PV cells were significantly decreased in fTSC2 KO mice overall compared to controls Figure 3C; Two-way RM ANOVA [F_geno_(1,17) = 10.51, *p* = 0.005; F_region_(9,153) = 9.479, *p* < 0.0001], they were particularly decreased in the middle subdivisions of the cortex (regions 3 and 4, *p* ≤ 0.019; 5–8, *p* < 0.01). SST cell densities were also decreased overall in fTSC2 KO mice Figure 3E; [F_geno_(1,17) = 5.330, *p* = 0.034; F_region_(9,153) = 8.299, *p* < 0.0001] but were particularly decreased in middle subdivisions (regions 4–6 and 8; *p*’s ≤ 0.041). Interestingly, the density of PV or SST interneurons did not correlate with seizure severity in epileptic mice, measured by seizures per day [Figure 3D, Pearson r correlation: (r-_PV_ = 0.3721, *p* = 0.324; r-_SST_ = -0.054, *p* = 0.890)]. This suggests that interneurons alone may not account for the seizure burden in this model.

During the inhibitory cell analyses, mCherry + PV and mCherry + SST cells were found in control and fTSC2 KO tissue. Although CaMKII is typically associated with excitatory neurons, previous reports indicate that some cortical inhibitory cells do express CaMKII (Keaveney et al., 2020), and viral expression in some inhibitory cells could be expected. Notably, mCherry + inhibitory cells in fTSC2 KO tissue had visibly larger somas, suggesting they are TSC2 deficient (example SST TSC2 KO cell in Figure 3A). In our samples, 5.754 ± 2.900% (control, *n* = 9) and 11.380 ± 2.963% (fTSC2 KO, *n* = 9) of all mCherry + cells were co-labeled with either PV or SST. The proportions did not differ between control and fTSC2 KO groups [t_PV_(16) = 0.724, *p* = 0.480; t_SST_(16) = 1.545, *p* = 0.142; t_PV_ _SST_(16) = 1.358, *p* = 0.193].

Despite a reduction in overall PV and SST cell densities in the fTSC2 KO tissue, the percentage of PV cells that expressed mCherry in control (5.119 ± 2.973, *n* = 10) and fTSC2 KO tissue (10.74 ± 4.158, *n* = 9) were comparable [t(17) = 1.116, *p* = 0.280]. In contrast, the percentage of SST cells that expressed mCherry was increased in fTSC2 KO tissue (11.34 ± 3.935) compared to the controls [3.022 ± 0.955; t(17) = 2.157, *p* = 0.046]. These data raise the possibility that *Tsc2* loss in inhibitory cells, particularly in SST cells, could render them less vulnerable to phenotypic loss or cell death than their wildtype counterparts.

### 3.4 Increased inflammatory response in the cortex of fTSC2 KO mice

**FIGURE 4:**
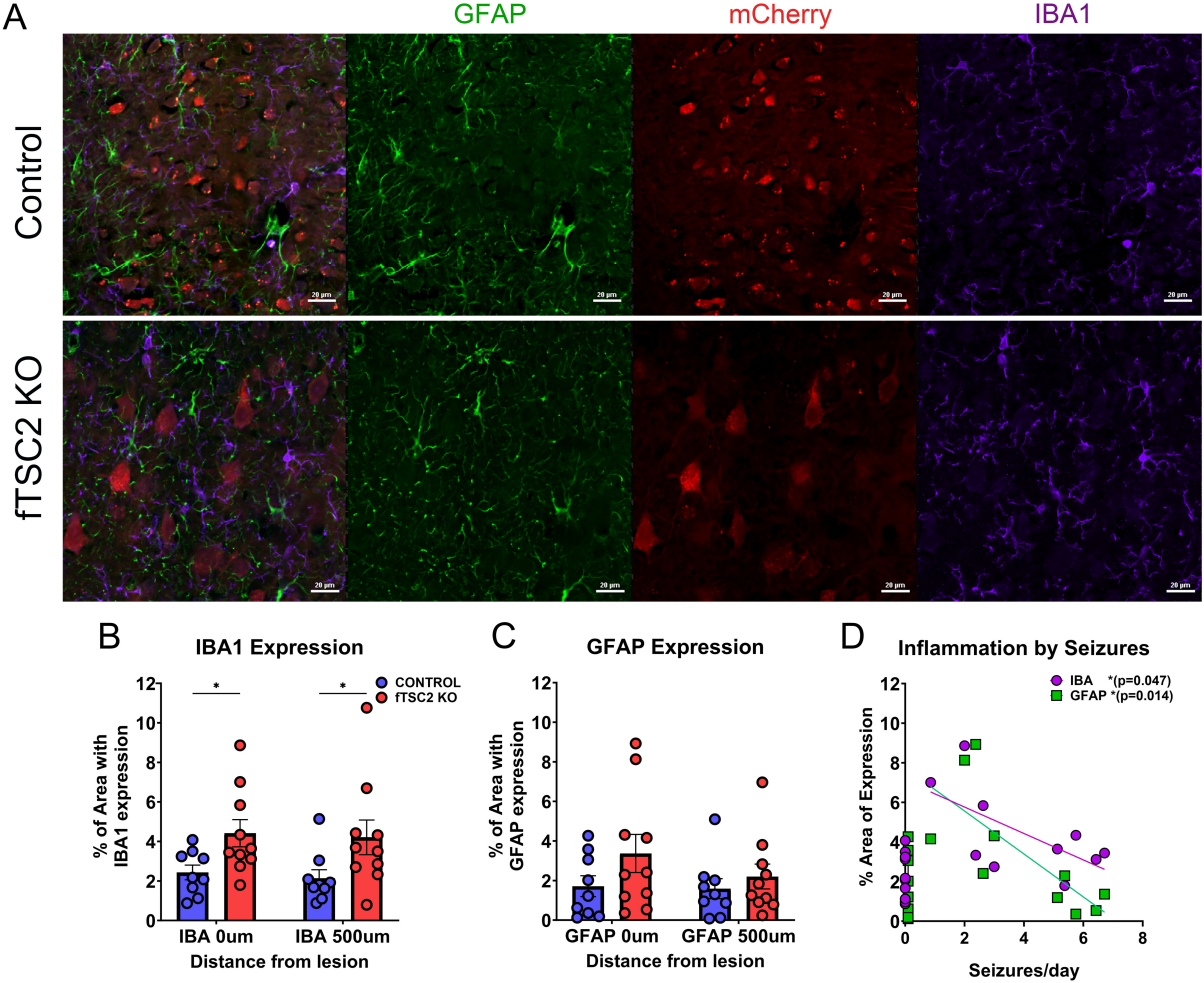
Microglia, but not astrocytes, were increased in focal *Tsc2* (fTSC2) knockout mice. **(A)** Tissue from control and focal *Tsc2* knockout (fTSC2 KO) mice was labeled with antibodies for glial fibrillary acidic protein (GFAP) and Iba1. Scale bars = 100 μm (60X). **(B)** At the lesion and up to 500 μm away from the center, ionized calcium-binding adapter molecule 1 (IBA1) expression was increased in fTSC2 KO tissue compared to control tissue (*p* = 0.03). **(C)** GFAP expression had a comparable level throughout control and fTSC2 KO tissue. **(D)** Average seizures per day was negatively correlated with both IBA1 (*r* = 0.638; *p* = 0.047) and GFAP expression (*r* = –0.742, *p* = 0.014) in fTSC2 KO mice. *, *p* < 0.05.

Inflammation has long been correlated with epilepsy (Ravizza et al., 2024). Activation of microglia and astrocytes is a hallmark of neuroinflammation and often precedes the onset of neuropathological symptoms (for review Sanz and Garcia-Gimeno, 2020). Upon activation, microglia can either promote resolution of the inflammatory response or contribute to chronic inflammation (Heneka et al., 2014). Similarly, astrocytes modulate neuroinflammation by secreting cytokines and chemokines that fine-tune the immune response (Rossi, 2015). Careful examination of ionized calcium-binding adapter molecule 1 (IBA1), expressed by microglia, and glial fibrillary acidic protein (GFAP), expressed by astrocytes, revealed no overlap with mCherry fluorescence (Figure 4A). This suggests that these non-neuronal cell types did not express Cre recombinase and remained unaffected by the virus in this model. To evaluate whether fTSC2 KO mice exhibited an inflammatory response, immunohistochemical markers of microglial (IBA1) and astrocytic (GFAP) activation were analyzed.

IBA1 expression was assessed both at the lesion site surrounding mCherry + cells and at a distance of 500 μm from the lesion to determine whether an inflammatory response occurred, and if so, whether it was localized to the lesion or more broadly distributed throughout the surrounding tissue. A more generalized pattern of inflammation could reflect the seizure phenotype rather than being driven by changes at the lesion site. In control tissue (Figure 4A), microglia exhibited a ramified morphology, characterized by long, thin branching processes and a small cell body, features consistent with an inactive state (Lier et al., 2021). Following an injury or other stimulation, microglia typically transition to a reactive phenotype, with shorter, thicker processes, an enlarged cell body, and increased branching. To quantify changes, an object-based area analysis was used to measure the total area of IBA1 + cells and their processes and to calculate the percentage of tissue occupied IBA1 expression. This method captured changes in microglial density and morphology, rather than changes in expression intensity per cell. Analyses revealed that the percentage of tissue occupied by IBA1 immunoreactive structures was increased in fTSC2 KO mice (*n* = 10) compared to controls (*n* = 9) at both the lesion core and 500 μm away Figure 4B; Two-way RM ANOVA, IBA1 [F_geno_(1,17) = 5.603, *p* = 0.030; F_distance_(1,17) = 0.577, *p* = 0.458], indicating enhanced microglial activation. Thus, this activation may be caused or exacerbated by seizure activity rather than being solely attributable to the targeted cellular manipulation.

Astrocytes become hypertrophic and upregulate GFAP expression when activated (Yang and Wang, 2015). To assess astrocytic activation, GFAP levels were measured in both control (*n* = 9) and fTSC2 KO tissue (*n* = 10). As shown in Figure 4A, average GFAP expression was comparable across genotypes and distances from the lesion Figure 4C; Two-way RM ANOVA, [F_geno_(1,17) = 1.569, *p* = 0.2273; F_distance_(1,17) = 2.719, *p* = 0.1175], indicating no significant astrocyte response. These findings suggest that, unlike microglia, astrocytes may not be broadly activated in fTSC2 KO mice.

A Pearson r correlation was used to investigate if either inflammation marker was related to seizures in individual mice. Indeed, average seizures per day was negatively correlated with both IBA1 (Figure 4D; *r* = −0.638; *p* = 0.047) and GFAP expression (*r* = −0.742, *p* = 0.014) in fTSC2 KO mice, such that higher seizures numbers were correlated with decreased IBA1 and GFAP activation. fTSC2 KO mice with higher seizure numbers had IBA1 and GFAP expression in the range of control mice, suggesting that mice with more severe epilepsy had less inflammation compared to those with fewer seizures per day.

### 3.5 fTSC2 KO mice display thigmotaxic behavior

Control/Td + and fTSC2 KO/Td + mice (injected with 1:10 AAV-mCherry-T2A-Cre/AAV-eGFP) were placed alone in novel circular cages, in a new room, and recorded through a top-mounted camera for 30 min to investigate their exploratory behavior. Of note, no animals had seizures during testing, although 2 fTSC2 KO/Td + mice had behavioral seizures within one day of testing (noted by the investigator). A volumetric analysis of the cortex showed no change in these fTSC2 KO/Td + mice compared to controls/Td + control/Td + , *n* = 7: 26.765 ± 1.781 mm^3^; fTSC2 KO/Td + , *n* = 9: 30.344 ± 1.549 mm^3^; *t*-test [t(14) = 1.519, *p* = 0.151]. Of note, the percentage of the cortex with infected cells (lesion size) was consistent between fTSC2 KO mice that underwent EEG or behavioral testing EEG, *n* = 10: 39.165 ± 1.810%; behavior, *n* = 9: 30.899 ± 5.207%; *t*-test [t(17) = 1.565, *p* = 0.136], even though less Cre + viral particles were injected.

**FIGURE 5:**
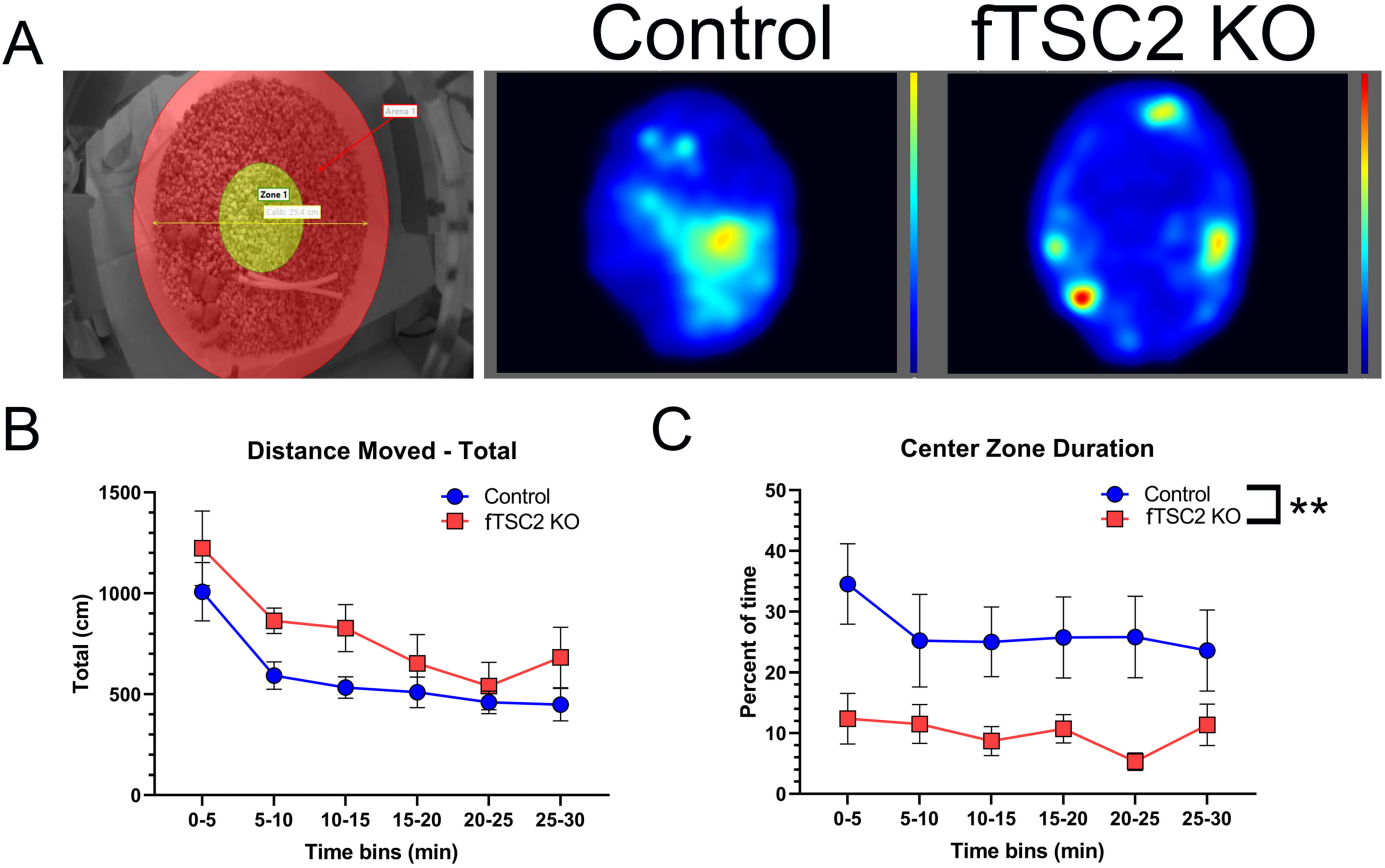
Focal *Tsc2* knockout (fTSC2 KO) mice display thigmotaxic behavior. **(A)** Control and fTSC2 KO mice spent 30 min in an open field, consisting of a circular cage with a 10-inch diameter and 8-inch wall height. The center zone (left image, yellow area) was defined as the middle 4-inch of the field. Animals were tracked and analyzed using EthoVision XT (Noldus Information Technology). Group heat maps show the average locations where control and fTSC2 KO mice spent their time. **(B)** The distance traveled in the open field was split into 5 min bins. Control and fTSC2 KO mice spent more time exploring the open field in the first 5 min, but their distance traveled was comparable. **(C)** fTSC2 KO mice spent less time in the center zone when compared with control mice. **, *p* < 0.01.

Control/Td + (*n* = 8) and fTSC2 KO/Td + (*n* = 9) mice moved similar distances in the cage during the 30 min period Figure 5B; Two-way RM ANOVA [F_group_(1,15) = 3.055, *p* = 0.101; F_distance_(5,75) = 12.661, *p* < 0.001]. As expected, mice from both groups traveled further in the first five minutes compared to all other time bins (Bonferroni *t*-tests, all *p*’s < 0.001), after which distances traveled during bins were comparable. These data show that both groups spent a comparable amount of time exploring the new environment, traveling the most when first placed in the new cage, and that neither group had a mobility or motivational issue.

Of interest, the location where control/Td + and fTSC2 KO/Td + mice spent their time differed between groups. fTSC2 KO/Td + mice spent less time in the center zone compared to controls during all time bins Figure 5C; Two-way RM ANOVA [F_group_(1,15) = 9.158, *p* = 0.009; F_time_(5,75) = 1.293, *p* = 0.276]. In a visual representation, group heat maps illustrate the propensity of fTSC2 KO/Td + mice to remain along the walls, whereas the control mice had more activity in the center zone (Figure 5A, generated by EthoVision XT software). The fTSC2 KO/Td + mice barely explored the center zones (9.985% ± 3.779% time in center), mostly moving around the outer edge, while the controls/Td + explored the center area of the novel cage approximately a quarter of the time (26.657% ± 4.008%).

## 4 Discussion

*TSC2* mutations have been associated with abnormal cell growth, intellectual disabilities, and neuropsychological disorders in patients, with 80%–90% having epilepsy (Holmes and Stafstrom, 2007). Here we show that focal lesions containing TSC2 KO cells are sufficient to *cause* epilepsy. In addition to recapitulating the seizure phenotype seen in TSC patients, histological changes in our fTSC2 KO mice largely model the disorder. TSC2 KO cells in this model are enlarged and have increased pS6 expression, while the lesions exhibit decreased inhibitory cell density and have increased microglia activation that extends outside of the lesion. Further, analysis of exploratory behavior indicated that TSC2 KO cells are sufficient to cause behavioral changes in the mice, namely anxiety-associated thigmotaxic behavior. This novel fTSC2 KO model allows for future studies to investigate the underlying mechanisms of epileptogenesis and behavioral deficits associated with TSC, as well as characterize how the lesion size and location can impact seizure severity and cognitive consequences.

### 4.1 Model comparison

In comparison to other TSC mouse models, our fTSC2 KO mice had smaller, focal cortical lesions, likely from a combination of both timing and injection target. Excitatory cells reach their final position during their first week of life (Kast and Levitt, 2019). In our postnatal day 2 injection model, knockout cells were likely near their final location before loss of the targeted protein; future experiments will focus on characterizing how cortical network development proceeds following viral infection in this model. In contrast, IUE models target cells undergoing development and migration, leading to a larger spread of abnormal neurons. Second, our injections targeted the frontal cortex, where the virus would affect a focal area with a heterogenous population of excitatory and inhibitory neurons. The *Tsc1* KO model by Prabhakar et al. (2013) also used a neonatal viral injection to initiate gene deletion at the same developmental time, but their intraventricular injections produced a scattering of abnormal cells. IUE manipulations, which target radial glial cells, can impact both neurons and glial cells and often cause large numbers of abnormal cells to span a cortical layer (Nguyen and Bordey, 2021). Altogether, our model targeted a small area of neurons, after most cortical development occurred, to create a focal lesion capable of generating seizures.

Our fTSC2 KO mice have a comparable phenotype to some IUE models of TSC but with fewer technical challenges. IUE models have been effective at manipulating specific neural populations by targeting neurons on specific embryonic days. Manipulations on embryonic day 14–15 impacted layer II/III principal cells, the same neurons thought to be severely affected during malformation of cortical development (Blümcke et al., 2009; D’Gama and Walsh, 2018). In each of these cases, animals went on to develop abnormal cells, with seizures beginning at 3–7 weeks (Hsieh et al., 2016; Lim et al., 2017; Reijnders et al., 2017); these mice typically have a normal lifespan (Nguyen and Bordey, 2022). Interestingly, a model of Rheb*^CA^*-electroporated mice had a similar seizure phenotype (approximately 5.7 seizures per day with mean duration of 39.4 s) (Hsieh et al., 2016) compared to our fTSC2 KO mice (5.3 seizures per day with mean duration of 42.2 s). Of note, our model did not have any evidence of mislamination associated with TSC, and abnormal cells spanned more cortical layers than the timed IUE models. However, IUE is challenging and can face low transfection efficiency, variability issues, and low pup survival rates (Nguyen and Bordey, 2022). Viral neonatal injections can be quick (a litter can take less than an hour to inject) and few to no mice are lost from the injection (Kim et al., 2014).

### 4.2 Epilepsy phenotype

Our fTSC2 KO model recapitulated the seizure phenotype of TSC patients, with less severe epilepsy and mortality than some other mouse models. Mice with a neonatal, intraventricular viral knockout of *Tsc1* displayed seizures within 2–3 weeks of age and had a mean survival age of only 66.5 days (Prabhakar et al., 2013). Other TSC models also have a severe phenotype, with seizures starting as young as 10 days post-gene deletion and early mortality spanning from 20 days post-gene deletion to under 8 weeks old (Wilson et al., 2005; Meikle et al., 2007; Way et al., 2009; Zeng et al., 2011; Prabhakar et al., 2013; Lim et al., 2017; Koene et al., 2019). In comparison, our fTSC2 KO mice exhibited robust seizures at 8 weeks of age when EEG recording started, suggesting seizures began at younger ages. Although we only recorded from mice for 7 days, we recorded from mice from ages 8 to 18 weeks old, and all fTSC2 KO mice had seizures. This data suggests that seizures would continue after the recording period, although future studies will be needed to assess longer-term seizure dynamics to determine if they would change in frequency or intensity. Importantly, over two-thirds of fTSC2 KO EEG mice survived until tissue harvest at up to 20 weeks old. This increased life span of fTSC2 KO mice would allow time to delineate mechanisms underlying epileptogenesis and allow for manipulations to investigate possible treatments.

### 4.3 Inhibitory neuron loss

Loss of or weakened inhibition has been detected in both patients and animal models of TSC and mTOR hyperactivation (Valencia et al., 2006; Talos et al., 2012; Bateup et al., 2013a; Zhong et al., 2021; Scheper et al., 2024). Here, we focused our investigation on PV and SST interneurons, two populations that are essential for cortical circuits and are known to be impacted in patients (Valencia et al., 2006; Scheper et al., 2024). PV and SST are found throughout layers II through IV, with both synapsing onto excitatory pyramidal cells (Xu et al., 2010; Naka and Adesnik, 2016). Our model showed significant PV loss in layers II/III to IV and SST loss primarily in deeper regions resembling layer V, consistent with previously described laminar distributions (Wall et al., 2016; Zhong et al., 2021). While our model results in less viral infection near the cortical edge, the loss of PV and SST was not proportional to the density of mCherry + cells; SST cell density reductions were most prominent in the mid-to-deeper cortical layers. These results are consistent with many other animal models of mTOR hyperactivation and spontaneous seizures that have also reported interneuron loss, including IUE of PIK3CA with hyperactive mTOR in the cortex (Zhong et al., 2021) and PTEN loss in dentate granule cells (LaSarge et al., 2021), although not all report this finding (Hsieh et al., 2016).

Our data lead us to question if interneuron loss is (1) a step in the epileptogenic process of our model, (2) a consequence of seizure activity, occurring once a threshold number of inhibitory cells are lost, or (3) due to failed migration of inhibitory cells to their appropriate cortical locations. Interestingly, interneuron loss preceded seizure activity in the IUE of PIK3CA model (Zhong et al., 2021). If inhibitory cell loss has a threshold, then the lack of correlation between seizure number and inhibitory density in our mice may indicate that they are past the threshold. Unfortunately, our study cannot disentangle the cause or timing of the interneuron loss. To determine whether interneuron loss contributes to seizures or is caused by them, future studies will investigate fTSC2 KO mice at the time of their first seizure. This would allow us to assess whether inhibitory cells are already absent, incorrectly localized, or expressing cell death markers. Additional experiments will also investigate TSC2 KO cells localized to a single hemisphere to better discriminate between the effects of epileptogenesis and seizure activity.

Of note, a small population of inhibitory cells were infected by our CaMKIIa virus. Previously, CaMKIIa was thought to be a specific promoter for excitatory cells (Liu and Jones, 1996). However, labeling via a miRNA-based viral gene targeting strategy recently showed that a small population of PV and SST interneurons in the cortex express CaMKIIa (Keaveney et al., 2020). Our model confirms this expression pattern, in which an average of 3% of SST and 5% of PV cells expressed mCherry in the control mice.

Our data suggests that *Tsc2* deletion may enhance the survivability of SST interneurons, as evidenced by the increased proportion of SST-mCherry + cells in our fTSC2 KO mice. There were no apparent changes in the phenotypic marker expression between the SST and PV cells, as the proportion of mCherry + SST and PV cells remained comparable between control and KO mice. Previous investigation from Malik et al. (2019) found that a subset of *Tsc1* deficient SSTs cells expressed PV and adopted the fast-spiking properties that are characteristic of that cell type, suggesting that mTOR signaling influences interneuron identity. A key distinction between the models is the timing of gene deletion. In our study, interneurons were post-mitotic and maturing at the time of *Tsc2* loss, whereas in the *Tsc1*-deficient SST model, deletion occurred during embryonic development. Our later timing likely limited the impact of *Tsc2* deletion on cell phenotypic expression, instead influencing cell size and survival. These findings highlight the importance of developmental timing and gene expression in shaping the interneuron population and the importance of further investigation into the distinct roles of *Tsc1* and *Tsc2* in interneuron development in TSC.

### 4.4 Inflammatory response

Gliosis and microglia reactivity were previously observed in patients and mouse models with mTOR hyperactivity, although our model only showed increased microglia activation. TSC patients have increased density and activation of microglia (Zimmer et al., 2020), especially in the lesion area and close to dysmorphic cells (Boer et al., 2008). Cortical tubers typically have an increased expression of GFAP and higher numbers of astrocytes in patients (Binder and Steinhäuser, 2021). Our fTSC2 KO mice recapitulate the patient lesion phenotype, showing increased microglia activation without GFAP labeling, highlighting a selective inflammatory response.

Inflammation has been closely linked to seizure activity in mouse models (Feliciano et al., 2011; Nguyen et al., 2019). Here, fTSC2 KO mice with *lower* seizure frequency had increased inflammation, outside the range of controls and at higher levels than mice with a higher seizure burden. This correlation leads to multiple possibilities. One is that a low number of seizures may trigger inflammation, while chronic epilepsy with high seizure burden could lead to dysregulation. Previous research has suggested that sustained brain inflammation becomes dysregulated in chronic epilepsy (Ravizza et al., 2024). Additionally, inflammation may be influenced or triggered by external factors such as our surgical intervention. Post-surgical inflammation could confound our interpretations of seizure-related inflammatory changes, although both the controls and fTSC2 KO mice underwent similar surgeries. Another possibility is that epileptogenic changes, like cell loss, occurred leading up to the chronic seizure phenotype, after which the inflammatory system could have reached a new homeostatic state. Our fTSC2 KO model lacked a correlation between inhibitory cell loss and seizure number. As discussed, inhibitory cell loss could have happened during epileptogenesis or during the initiation of seizures. In either possibility, gliosis and microglia activation could be triggered in connection with secondary changes accompanying epileptogenesis, to include cell death, and then decrease when vulnerable cells are gone. Future investigation into the timing of inflammation, cell loss, and epileptogenesis may shed insight into the cascade of events that occurs during epileptogenesis through to the chronic stage, potentially informing therapeutic strategies for early intervention.

### 4.5 Thigmotaxic, anxiety-like behavior

TSC patients often have co-morbid neurological and psychological disorders; known as tuberous sclerosis-associated neuropsychiatric disorders [TAND, (for review see Marcinkowska et al., 2023)], these disorders include autism spectrum disorders, intellectual disability, depression, and anxiety (Kingswood et al., 2017; for review, Santos et al., 2025). The multidisciplinary study of TSC, the TuberOus SClerosis registry to increase disease Awareness (TOSCA), reported 9.7% of TSC patients were diagnosed with an anxiety disorder (Kingswood et al., 2017; Marcinkowska et al., 2023). Like the patients, mouse models of TSC have shown varying anxiety phenotypes along a spectrum that corresponds to the *Tsc2* genotype. Here, the fTSC2 KO lesions in frontal cortex led to anxiety-related thigmotaxic behavior, or wall hugging, in the mice without any gross motor dysfunction or motivation deficits. Thigmotaxis is widely considered an indicator of anxiety or predator avoidance (Hall and Ballachey, 1932; Seibenhener and Wooten, 2015; Zhang et al., 2023). In addition, the tendency to show thigmotaxic behavior gradually decreases over time (Simon et al., 1994).

Anxious behaviors have been observed in multiple rodent models of TSC. *Tsc2* dominant negative (*Tsc2*-DN) mice, which have a mutation in the c-terminus that leads to Rheb activation, have exhibited an increase in anxiety-related behaviors (Ehninger and Silva, 2011; Chévere-Torres et al., 2012). Our mice behaved similarly to *Tsc2*-DN mice, which had decreased center exploration and traveled a similar distance in an open field; *Tsc2*-DN mice also spent less time in the open arms of an elevated plus maze (EPM), revealing anxiety-like behavior (Ehninger and Silva, 2011). Additionally, *Tsc2* haploinsufficient rats showed similar motor activity to controls in an open field but had less exploration (Waltereit et al., 2011). On the other side of the continuum, *Tsc2* ± mice performed comparably to controls in both open field and elevated plus maze mice in Ehninger et al. (2008); yet, removal of both copies of *Tsc2* was associated with anxiety-like behavior. Graded loss of tuberin in a mouse model of TSC corresponded to an anxiety phenotype that corresponded to the TSC2 protein level (Yuan et al., 2012). Data supports a mechanism for mTOR in TANDs that is separate from epilepsy. Further delineation of behavioral and cognitive deficits, and their relationship to lesion size and placement, could aid in understanding and treating neuropsychological disorders in TSC patients. Future experiments with our fTSC2 KO mouse model are designed to target brain regions with varying size lesions to accomplish this task.

## 5 Conclusion

In conclusion, deletion of *Tsc2* from neurons in a focal region of the cortex is sufficient to cause epilepsy. This model recapitulates the seizure phenotype that impacts most TSC patients, allowing for investigation into the epileptogenic process caused by this mutation. fTSC2 KO mice had abnormal cell growth, decreased inhibitory cell density, and increased microglia activation. Moreover, fTSC2 KO mice displayed an anxiety-like phenotype. Altogether, this model provides a means to investigate the link between TSC, seizures, neuropsychiatric disorders, and possible targets for therapeutic interventions.

## Data Availability

The raw data supporting the conclusions of this article will be made available by the authors, without undue reservation.

## References

[B1] Abs E. Goorden S. M. Schreiber J. Overwater I. E. Hoogeveen-Westerveld M. Bruinsma C. F. (2013). TORC1-dependent epilepsy caused by acute biallelic Tsc1 deletion in adult mice. *Ann. Neurol.* 74 569–579. 10.1002/ana.23943 23720219

[B2] Aronica E. Specchio N. Luinenburg M. J. Curatolo P. (2023). Epileptogenesis in tuberous sclerosis complex-related developmental and epileptic encephalopathy. *Brain* 146 2694–2710. 10.1093/brain/awad048 36806388 PMC10316778

[B3] Bateup H. S. Denefrio C. L. Johnson C. A. Saulnier J. L. Sabatini B. L. (2013a). Temporal dynamics of a homeostatic pathway controlling neural network activity. *Front. Mol. Neurosci.* 6:28. 10.3389/fnmol.2013.00028 24065881 PMC3776619

[B4] Bateup H. S. Johnson C. A. Denefrio C. L. Saulnier J. L. Kornacker K. Sabatini B. L. (2013b). Excitatory/inhibitory synaptic imbalance leads to hippocampal hyperexcitability in mouse models of tuberous sclerosis. *Neuron* 78 510–522. 10.1016/j.neuron.2013.03.017 23664616 PMC3690324

[B5] Binder D. K. Steinhäuser C. (2021). Astrocytes and epilepsy. *Neurochem. Res.* 46 2687–2695. 10.1007/s11064-021-03236-x 33661442

[B6] Bissler J. J. Kingswood J. C. Radzikowska E. Zonnenberg B. A. Belousova E. Frost M. D. (2017). Everolimus long-term use in patients with tuberous sclerosis complex: Four-year update of the EXIST-2 study. *PLoS One* 12:e0180939. 10.1371/journal.pone.0180939 28792952 PMC5549893

[B7] Blümcke I. Vinters H. V. Armstrong D. Aronica E. Thom M. Spreafico R. (2009). Malformations of cortical development and epilepsies: Neuropathological findings with emphasis on focal cortical dysplasia. *Epileptic Disord.* 11 181–193. 10.1684/epd.2009.0261 19736171

[B8] Boer K. Jansen F. Nellist M. Redeker S. van den Ouweland A. M. Spliet W. G. (2008). Inflammatory processes in cortical tubers and subependymal giant cell tumors of tuberous sclerosis complex. *Epilepsy Res.* 78 7–21. 10.1016/j.eplepsyres.2007.10.002 18023148

[B9] Chévere-Torres I. Maki J. M. Santini E. Klann E. (2012). Impaired social interactions and motor learning skills in tuberous sclerosis complex model mice expressing a dominant/negative form of tuberin. *Neurobiol. Dis.* 45 156–164. 10.1016/j.nbd.2011.07.018 21827857 PMC3225564

[B10] Choi Y. J. Di Nardo A. Kramvis I. Meikle L. Kwiatkowski D. J. Sahin M. (2008). Tuberous sclerosis complex proteins control axon formation. *Genes Dev.* 22 2485–2495. 10.1101/gad.1685008 18794346 PMC2546692

[B11] Chow D. K. Groszer M. Pribadi M. Machniki M. Carmichael S. T. Liu X. (2009). Laminar and compartmental regulation of dendritic growth in mature cortex. *Nat. Neurosci.* 12 116–118. 10.1038/nn.2255 19151711 PMC2842592

[B12] Curatolo P. Nabbout R. Lagae L. Aronica E. Ferreira J. C. Feucht M. (2018). Management of epilepsy associated with tuberous sclerosis complex: Updated clinical recommendations. *Eur. J. Paediatr. Neurol.* 22 738–748. 10.1016/j.ejpn.2018.05.006 29880258

[B13] Delia M. T. Delia M. T. Hongyu S. Béla K. Hongyu S. Bela K. (2012). Altered inhibition in tuberous sclerosis and type IIb cortical dysplasia. *Ann. Neurol*. 71 539–551. 10.1002/ana.22696 22447678 PMC3334406

[B14] D’Gama A. M. Walsh C. A. (2018). Somatic mosaicism and neurodevelopmental disease. *Nat. Neurosci.* 21 1504–1514. 10.1038/s41593-018-0257-3 30349109

[B15] Dusing M. LaSarge C. L. White A. Jerow L. G. Gross C. Danzer S. C. (2023). Neurovascular development in Pten and Tsc2 mouse mutants. *eNeuro* 10:ENEURO.0340-22.2023. 10.1523/ENEURO.0340-22.2023 36759189 PMC9953070

[B16] Ehninger D. Silva A. J. (2011). Increased levels of anxiety-related behaviors in a Tsc2 dominant negative transgenic mouse model of tuberous sclerosis. *Behav. Genet.* 41 357–363. 10.1007/s10519-010-9398-1 20882401 PMC3102774

[B17] Ehninger D. Han S. Shilyansky C. Zhou Y. Li W. Kwiatkowski D. J. (2008). Reversal of learning deficits in a Tsc2+/- mouse model of tuberous sclerosis. *Nat. Med.* 14 843–848. 10.1038/nm1788 18568033 PMC2664098

[B18] Feliciano D. M. Su T. Lopez J. Platel J.-C. Bordey A. (2011). Single-cell Tsc1 knockout during corticogenesis generates tuber-like lesions and reduces seizure threshold in mice. *J. Clin. Invest.* 121 1596–1607. 10.1172/jci44909 21403402 PMC3069783

[B19] Fingar D. C. Blenis J. (2004). Target of rapamycin (TOR): An integrator of nutrient and growth factor signals and coordinator of cell growth and cell cycle progression. *Oncogene* 23 3151–3171. 10.1038/sj.onc.1207542 15094765

[B20] Franz D. N. Budde K. Kingswood J. C. Belousova E. Sparagana S. De Vries P. J. (2018). Effect of everolimus on skin lesions in patients treated for subependymal giant cell astrocytoma and renal angiomyolipoma: Final 4-year results from the randomized EXIST-1 and EXIST-2 studies. *J. Eur. Acad. Dermatol. Venereol.* 32 1796–1803. 10.1111/jdv.14964 29569806

[B21] Fraser M. M. Zhu X. Kwon C.-H. Uhlmann E. J. Gutmann D. H. Baker S. J. (2004). Pten loss causes hypertrophy and increased proliferation of astrocytes in vivo. *Cancer Res.* 64 7773–7779. 10.1158/0008-5472.can-04-2487 15520182

[B22] French J. A. Lawson J. A. Yapici Z. Ikeda H. Polster T. Nabbout R. (2016). Adjunctive everolimus therapy for treatment-resistant focal-onset seizures associated with tuberous sclerosis (EXIST-3): A phase 3, randomised, double-blind, placebo-controlled study. *Lancet* 388 2153–2163. 10.1016/s0140-6736(16)31419-2 27613521

[B23] Hall C. Ballachey E. L. (1932). A study of the rat’s behavior in a field. A contribution to method in comparative psychology. *University of California Publications Psychol.* 6, 1–12.

[B24] Heneka M. T. Kummer M. P. Latz E. (2014). Innate immune activation in neurodegenerative disease. *Nat. Rev. Immunol.* 14 463–477. 10.1038/nri3705 24962261

[B25] Hernandez O. Way S. McKenna J. Gambello M. J. (2007). Generation of a conditional disruption of the Tsc2 gene. *Genesis* 45 101–106. 10.1002/dvg.20271 17245776

[B26] Holmes G. L. Stafstrom C. E. (2007). Tuberous sclerosis complex and epilepsy: Recent developments and future challenges. *Epilepsia* 48 617–630. 10.1111/j.1528-1167.2007.01035.x 17386056

[B27] Hsieh L. S. Wen J. H. Claycomb K. Huang Y. Harrsch F. A. Naegele J. R. (2016). Convulsive seizures from experimental focal cortical dysplasia occur independently of cell misplacement. *Nat. Commun.* 7:11753. 10.1038/ncomms11753 27249187 PMC4895394

[B28] Jobst B. C. Cascino G. D. (2015). Resective epilepsy surgery for drug-resistant focal epilepsy: A review. *JAMA* 313 285–293. 10.1001/jama.2014.17426 25602999

[B29] Kast R. J. Levitt P. (2019). Precision in the development of neocortical architecture: From progenitors to cortical networks. *Progr. Neurobiol*. 175 77–95. 10.1016/j.pneurobio.2019.01.003 30677429 PMC6402587

[B30] Keaveney M. K. Rahsepar B. Tseng H. A. Fernandez F. R. Mount R. A. Ta T. (2020). CaMKIIalpha-positive interneurons identified via a microRNA-based viral gene targeting strategy. *J. Neurosci.* 40 9576–9588. 10.1523/JNEUROSCI.2570-19.2020 33158963 PMC7726537

[B31] Kim J. Y. Grunke S. D. Levites Y. Golde T. E. Jankowsky J. L. (2014). Intracerebroventricular viral injection of the neonatal mouse brain for persistent and widespread neuronal transduction. *J. Vis. Exp.* 91:51863. 10.3791/51863 25286085 PMC4199253

[B32] Kingswood J. C. D’Augères G. B. Belousova E. Ferreira J. C. Carter T. Castellana R. (2017). TuberOus SClerosis registry to increase disease Awareness (TOSCA) – baseline data on 2093 patients. *Orphanet J. Rare Dis.* 12:2. 10.1186/s13023-016-0553-5 28057044 PMC5217262

[B33] Kobayashi T. Minowa O. Kuno J. Mitani H. Hino O. Noda T. (1999). Renal carcinogenesis, hepatic hemangiomatosis, and embryonic lethality caused by a germ-line Tsc2 mutation in mice. *Cancer Res.* 59 1206–1211. 10096549

[B34] Kobayashi T. Minowa O. Sugitani Y. Takai S. Mitani H. Kobayashi E. (2001). A germ-line Tsc1 mutation causes tumor development and embryonic lethality that are similar, but not identical to, those caused by Tsc2 mutation in mice. *Proc. Natl. Acad. Sci. U S A.* 98 8762–8767. 10.1073/pnas.151033798 11438694 PMC37509

[B35] Koene L. M. C. Van Grondelle S. E. Proietti Onori M. Wallaard I. Kooijman N. H. R. M. Van Oort A. (2019). Effects of antiepileptic drugs in a new TSC/mTOR-dependent epilepsy mouse model. *Ann. Clin. Transl. Neurol.* 6 1273–1291. 10.1002/acn3.50829 31353861 PMC6649373

[B36] Kwon C. H. Luikart B. W. Powell C. M. Zhou J. Matheny S. A. Zhang W. (2006b). Pten regulates neuronal arborization and social interaction in mice. *Neuron* 50 377–388. 10.1016/j.neuron.2006.03.023 16675393 PMC3902853

[B37] Kwon C. H. Zhu X. Zhang J. Knoop L. L. Tharp R. Smeyne R. J. (2001). Pten regulates neuronal soma size: A mouse model of Lhermitte-Duclos disease. *Nat. Genet.* 29 404–411. 10.1038/ng781 11726927

[B38] Kwon C.-H. Zhou J. Li Y. Kim K. W. Hensley L. L. Baker S. J. (2006a). Neuron-specific enolase-cre mouse line with cre activity in specific neuronal populations. *Genesis* 44 130–135. 10.1002/gene.20197 16496331

[B39] Lasarge C. L. Danzer S. C. (2014). Mechanisms regulating neuronal excitability and seizure development following mTOR pathway hyperactivation. *Front. Mol. Neurosci.* 7:18. 10.3389/fnmol.2014.00018 24672426 PMC3953715

[B40] LaSarge C. L. Pun R. Y. K. Gu Z. Riccetti M. R. Namboodiri D. V. Tiwari D. (2021). mTOR-driven neural circuit changes initiate an epileptogenic cascade. *Progr. Neurobiol.* 200:101974. 10.1016/j.pneurobio.2020.101974 33309800 PMC8026598

[B41] Lier J. Streit W. J. Bechmann I. (2021). Beyond activation: Characterizing microglial functional phenotypes. *Cells* 10:2236. 10.3390/cells10092236 34571885 PMC8464670

[B42] Lim J. S. Gopalappa R. Kim S. H. Ramakrishna S. Lee M. Kim W.-I. (2017). Somatic mutations in TSC1 and TSC2 cause focal cortical dysplasia. *Am. J. Hum. Genet.* 100 454–472. 10.1016/j.ajhg.2017.01.030 28215400 PMC5339289

[B43] Lim J. S. Kim W.-I. Kang H.-C. Kim S. H. Park A. H. Park E. K. (2015). Brain somatic mutations in MTOR cause focal cortical dysplasia type II leading to intractable epilepsy. *Nat. Med.* 21:395. 10.1038/nm.3824 25799227

[B44] Lin T. V. Hsieh L. Kimura T. Malone T. J. Bordey A. (2016). Normalizing translation through 4E-BP prevents mTOR-driven cortical mislamination and ameliorates aberrant neuron integration. *Proc. Natl. Acad. Sci. U S A.* 113 11330–11335. 10.1073/pnas.1605740113 27647922 PMC5056085

[B45] Litwa K. (2022). Shared mechanisms of neural circuit disruption in tuberous sclerosis across lifespan: Bridging neurodevelopmental and neurodegenerative pathology. *Front. Genet.* 13:997461. 10.3389/fgene.2022.997461 36506334 PMC9732432

[B46] Liu G. Slater N. Perkins A. (2017). Epilepsy: Treatment options. *Am. Fam. Physician* 96 87–96. 28762701

[B47] Liu X. B. Jones E. G. (1996). Localization of alpha type II calcium calmodulin-dependent protein kinase at glutamatergic but not gamma-aminobutyric acid (GABAergic) synapses in thalamus and cerebral cortex. *Proc. Natl. Acad. Sci.* 93 7332–7336. 10.1073/pnas.93.14.7332 8692993 PMC38984

[B48] Ma X. M. Blenis J. (2009). Molecular mechanisms of mTOR-mediated translational control. *Nat. Rev. Mol. Cell Biol.* 10 307–318. 10.1038/nrm2672 19339977

[B49] Magri L. Cambiaghi M. Cominelli M. Alfaro-Cervello C. Cursi M. Pala M. (2011). Sustained activation of mTOR pathway in embryonic neural stem cells leads to development of tuberous sclerosis complex-associated lesions. *Cell. Stem Cell.* 9 447–462. 10.1016/j.stem.2011.09.008 22056141

[B50] Malik R. Pai E. L.-L. Rubin A. N. Stafford A. M. Angara K. Minasi P. (2019). Tsc1 represses parvalbumin expression and fast-spiking properties in somatostatin lineage cortical interneurons. *Nat. Commun.* 10:4994. 10.1038/s41467-019-12962-4 31676823 PMC6825152

[B51] Marcinkowska A. B. Tarasewicz A. Jóźwiak S. Dębska-Ślizień A. Szurowska E. (2023). Tuberous sclerosis complex-associated neuropsychiatric disorders. *Psychiatria Polska* 57 823–842. 10.12740/pp/onlinefirst/146265 36370437

[B52] Marcotte L. Crino P. B. (2006). The neurobiology of the tuberous sclerosis complex. *NeuroMolecular Med.* 8 531–546. 10.1385/NMM:8:4:531 17028374

[B53] Marino S. Krimpenfort P. Leung C. van der Korput H. A. G. M. Trapman J. Camenisch I. (2002). PTEN is essential for cell migration but not for fate determination and tumourigenesis in the cerebellum. *Development* 129 3513–3522. 10.1242/dev.129.14.3513 12091320

[B54] Marsan E. Baulac S. (2018). Review: Mechanistic target of rapamycin (mTOR) pathway, focal cortical dysplasia and epilepsy. *Neuropathol. Appl. Neurobiol.* 44 6–17. 10.1111/nan.12463 29359340

[B55] Meikle L. Pollizzi K. Egnor A. Kramvis I. Lane H. Sahin M. (2008). Response of a neuronal model of tuberous sclerosis to mammalian target of rapamycin (mTOR) inhibitors: Effects on mTORC1 and Akt signaling lead to improved survival and function. *J. Neurosci.* 28 5422–5432. 10.1523/jneurosci.0955-08.2008 18495876 PMC2633923

[B56] Meikle L. Talos D. M. Onda H. Pollizzi K. Rotenberg A. Sahin M. (2007). A mouse model of tuberous sclerosis: Neuronal loss of Tsc1 causes dysplastic and ectopic neurons, reduced myelination, seizure activity, and limited survival. *J. Neurosci.* 27 5546–5558. 10.1523/jneurosci.5540-06.2007 17522300 PMC6672762

[B57] Naka A. Adesnik H. (2016). Inhibitory circuits in cortical layer 5. *Front. Neural Circuits* 10:35. 10.3389/fncir.2016.00035 27199675 PMC4859073

[B58] Napolioni V. Moavero R. Curatolo P. (2009). Recent advances in neurobiology of tuberous sclerosis complex. *Brain Dev.* 31 104–113. 10.1016/j.braindev.2008.09.013 19028034

[B59] National Research Council (US) Committee for the Update of the Guide for the Care and Use of Laboratory Animals. (2011). *Guide for the Care and Use of Laboratory Animals, 8th Edn*. Washington, DC: National Academies Press (US). 10.17226/12910 21595115

[B60] Nguyen L. H. Bordey A. (2021). Convergent and divergent mechanisms of epileptogenesis in mTORopathies. *Front. Neuroanat.* 15:664695. 10.3389/fnana.2021.664695 33897381 PMC8064518

[B61] Nguyen L. H. Bordey A. (2022). Current review in basic science: Animal models of focal cortical dysplasia and epilepsy. *Epilepsy Curr.* 22 234–240. 10.1177/15357597221098230 36187145 PMC9483763

[B62] Nguyen L. H. Mahadeo T. Bordey A. (2019). mTOR hyperactivity levels influence the severity of epilepsy and associated neuropathology in an experimental model of tuberous sclerosis complex and focal cortical dysplasia. *J. Neurosci.* 39 2762–2773. 10.1523/jneurosci.2260-18.2019 30700531 PMC6445990

[B63] Onda H. Lueck A. Marks P. W. Warren H. B. Kwiatkowski D. J. (1999). Tsc2(+/-) mice develop tumors in multiple sites that express gelsolin and are influenced by genetic background. *J. Clin. Invest.* 104 687–695. 10.1172/jci7319 10491404 PMC408440

[B64] Onori M. Martina Proietti O. Koene L. Linda M. C. K. Schafer C. Mark N. (2020). RHEB/mTOR-hyperactivity causing cortical malformations drives seizures through increased axonal connectivity. *bioRxiv [Preprint]* 10.1101/2020.07.08.189399

[B65] Prabhakar S. Goto J. Zuang X. Sena-Esteves M. Bronson R. Brockmann J. (2013). Stochastic model of Tsc1 lesions in mouse brain. *PLoS One* 8:e64224. 10.1371/journal.pone.0064224 23696872 PMC3655945

[B66] Ravizza T. Scheper M. Di Sapia R. Gorter J. Aronica E. Vezzani A. (2024). mTOR and neuroinflammation in epilepsy: Implications for disease progression and treatment. *Nat. Rev. Neurosci.* 25 334–350. 10.1038/s41583-024-00805-1 38531962

[B67] Reijnders M. R. F. Kousi M. Van Woerden G. M. Klein M. Bralten J. Mancini G. M. S. (2017). Variation in a range of mTOR-related genes associates with intracranial volume and intellectual disability. *Nat. Commun.* 8:1052. 10.1038/s41467-017-00933-6 29051493 PMC5648772

[B68] Rosser T. Panigrahy A. McClintock W. (2006). The diverse clinical manifestations of tuberous sclerosis complex: A review. *Semin. Pediatr. Neurol.* 13 27–36. 10.1016/j.spen.2006.01.008 16818173

[B69] Rossi D. (2015). Astrocyte physiopathology: At the crossroads of intercellular networking, inflammation and cell death. *Prog. Neurobiol.* 130 86–120. 10.1016/j.pneurobio.2015.04.003 25930681

[B70] Rout P. Thomas A. (2025). “*Tuberous sclerosis [Updated 2025 Jun 2]*,” in StatPearls [Internet]. Treasure Island, FL: StatPearls Publishing. Available online at: https://www.ncbi.nlm.nih.gov/books/NBK538492/30860727

[B71] Santos V. R. Jerow L. G. LaSarge C. L. (2025). Behavioral analyses in rodent models of tuberous sclerosis complex. *Epilepsy Behav.* 165:110313. 10.1016/j.yebeh.2025.110313 39978075

[B72] Sanz P. Garcia-Gimeno M. A. (2020). Reactive glia inflammatory signaling pathways and epilepsy. *Int. J. Mol. Sci.* 21:4096. 10.3390/ijms21114096 32521797 PMC7312833

[B73] Sasongko T. H. Kademane K. Chai Soon, Hou S. Jocelyn T. X. Zabidi-Hussin Z. (2023). Rapamycin and rapalogs for tuberous sclerosis complex. *Cochrane Database Syste. Rev.* 7:CD011272. 10.1002/14651858.CD011272.pub3 37432030 PMC10334695

[B74] Scheper M. Sørensen F. N. F. Ruffolo G. Gaeta A. Lissner L. J. Anink J. J. (2024). Impaired GABAergic regulation and developmental immaturity in interneurons derived from the medial ganglionic eminence in the tuberous sclerosis complex. *Acta Neuropathol.* 147:80. 10.1007/s00401-024-02737-7 38714540 PMC11076412

[B75] Seibenhener M. L. Wooten M. C. (2015). Use of the open field maze to measure locomotor and anxiety-like behavior in mice. *J. Vis. Exp.* 96:e52434. 10.3791/52434 25742564 PMC4354627

[B76] Simon P. Dupuis R. Costentin J. (1994). Thigmotaxis as an index of anxiety in mice. Influence of dopaminergic transmissions. *Behav. Brain Res.* 61 59–64. 10.1016/0166-4328(94)90008-6 7913324

[B77] Specchio N. Pavia G. C. De Palma L. De Benedictis A. Pepi C. Conti M. (2022). Current role of surgery for tuberous sclerosis complex-associated epilepsy. *Pediatr. Invest.* 6 16–22. 10.1002/ped4.12312 35382422 PMC8960933

[B78] Talos D. M. Sun H. Zhou X. Fitzgerald E. C. Jackson M. C. Klein P. M. (2012). The interaction between early life epilepsy and autistic-like behavioral consequences: A role for the mammalian target of rapamycin (mTOR) pathway. *PLoS One* 7:e35885. 10.1371/journal.pone.0035885 22567115 PMC3342334

[B79] Tarkowski B. Kuchcinska K. Blazejczyk M. Jaworski J. (2019). Pathological mTOR mutopment. *Hum. Mol. Genet.* 28 2107–2119. 10.1093/hmg/ddz042 30789219

[B80] Tsai V. Parker W. E. Orlova K. A. Baybis M. Chi A. W. Berg B. D. (2014). Fetal brain mTOR signaling activation in tuberous sclerosis complex. *Cereb. Cortex* 24 315–327. 10.1093/cercor/bhs310 23081885 PMC3888364

[B81] Valencia I. Legido A. Yelin K. Khurana D. Kothare S. V. Katsetos C. D. (2006). Anomalous inhibitory circuits in cortical tubers of human tuberous sclerosis complex associated with refractory epilepsy: Aberrant expression of parvalbumin and calbindin-D28k in dysplastic cortex. *J. Child Neurol.* 21 1058–1063. 10.1177/7010.2006.00242 17156698

[B82] Wall N. R. De La Parra M. Sorokin J. M. Taniguchi H. Huang Z. J. Callaway E. M. (2016). Brain-wide maps of synaptic input to cortical interneurons. *J. Neurosci.* 36 4000–4009. 10.1523/jneurosci.3967-15.2016 27053207 PMC4821911

[B83] Waltereit R. Japs B. Schneider M. de Vries P. J. Bartsch D. (2011). Epilepsy and Tsc2 haploinsufficiency lead to autistic-like social deficit behaviors in rats. *Behav. Genet.* 41 364–372. 10.1007/s10519-010-9399-0 20927644

[B84] Wang Y. Greenwood J. S. Calcagnotto M. E. Kirsch H. E. Barbaro N. M. Baraban S. C. (2007). Neocortical hyperexcitability in a human case of tuberous sclerosis complex and mice lacking neuronal expression of TSC1. *Ann. Neurol.* 61 139–152. 10.1002/ana.21058 17279540

[B85] Wataya-Kaneda M. Ohno Y. Fujita Y. Yokozeki H. Niizeki H. Ogai M. (2018). Sirolimus gel treatment vs placebo for facial angiofibromas in patients with tuberous sclerosis complex. *JAMA Dermatol.* 154:781. 10.1001/jamadermatol.2018.1408 29800026 PMC6128500

[B86] Way S. W. McKenna J. Mietzsch U. Reith R. M. Wu H. C. Gambello M. J. (2009). Loss of Tsc2 in radial glia models the brain pathology of tuberous sclerosis complex in the mouse. *Hum. Mol. Genet.* 18 1252–1265. 10.1093/hmg/ddp025 19150975 PMC2655769

[B87] Wilson C. Idziaszczyk S. Parry L. Guy C. Griffiths D. F. Lazda E. (2005). A mouse model of tuberous sclerosis 1 showing background specific early post-natal mortality and metastatic renal cell carcinoma. *Hum. Mol. Genet.* 14 1839–1850. 10.1093/hmg/ddi190 15888477

[B88] Xu X. Roby K. D. Callaway E. M. (2010). Immunochemical characterization of inhibitory mouse cortical neurons: Three chemically distinct classes of inhibitory cells. *J. Compar. Neurol.* 518 389–404. 10.1002/cne.22229 19950390 PMC2804902

[B89] Yamashiro K. Ikegaya Y. Matsumoto N. (2022). In utero electroporation for manipulation of specific neuronal populations. *Membranes* 12:513. 10.3390/membranes12050513 35629839 PMC9147339

[B90] Yang Z. Wang K. K. (2015). Glial fibrillary acidic protein: From intermediate filament assembly and gliosis to neurobiomarker. *Trends Neurosci.* 38 364–374. 10.1016/j.tins.2015.04.003 25975510 PMC4559283

[B91] Yuan E. Tsai P. T. Greene-Colozzi E. Sahin M. Kwiatkowski D. J. Malinowska I. A. (2012). Graded loss of tuberin in an allelic series of brain models of TSC correlates with survival, and biochemical, histological and behavioral features. *Hum. Mol. Genet.* 21 4286–4300. 10.1093/hmg/dds262 22752306 PMC3441124

[B92] Yue Q. Groszer M. Gil J. S. Berk A. J. Messing A. Wu H. (2005). PTEN deletion in Bergmann glia leads to premature differentiation and affects laminar organization. *Development* 132 3281–3291. 10.1242/dev.01891 15944184

[B93] Zaroff C. M. Barr W. B. Carlson C. Lajoie J. Madhavan D. Miles D. K. (2006). Mental retardation and relation to seizure and tuber burden in tuberous sclerosis complex. *Seizure* 15 558–562. 10.1016/j.seizure.2006.06.010 16935530

[B94] Zeng L.-H. Rensing N. R. Zhang B. Gutmann D. H. Gambello M. J. Wong M. (2011). Tsc2 gene inactivation causes a more severe epilepsy phenotype than Tsc1 inactivation in a mouse model of tuberous sclerosis complex. *Hum. Mol. Genet.* 20 445–454. 10.1093/hmg/ddq491 21062901 PMC3016907

[B95] Zhang L. Huang T. Teaw S. Nguyen L. H. Hsieh L. S. Gong X. (2020). Filamin A inhibition reduces seizure activity in a mouse model of focal cortical malformations. *Sci. Transl. Med.* 12:eaay0289. 10.1126/scitranslmed.aay0289 32075941 PMC12290962

[B96] Zhang X. Y. Diaz-delCastillo M. Kong L. Daniels N. MacIntosh-Smith W. Abdallah A. (2023). A systematic review and meta-analysis of thigmotactic behaviour in the open field test in rodent models associated with persistent pain. *PLoS One* 18:e0290382. 10.1371/journal.pone.0290382 37682863 PMC10490990

[B97] Zhong S. Zhao Z. Xie W. Cai Y. Zhang Y. Ding J. (2021). GABAergic interneuron and neurotransmission are mTOR-dependently disturbed in experimental focal cortical dysplasia. *Mol. Neurobiol.* 58 156–169. 10.1007/s12035-020-02086-y 32909150

[B98] Zhou J. Blundell J. Ogawa S. Kwon C.-H. Zhang W. Sinton C. (2009). Pharmacological inhibition of mTORC1 suppresses anatomical, cellular, and behavioral abnormalities in neural-specific Pten knock-out mice. *J. Neurosci.* 29 1773–1783. 10.1523/JNEUROSCI.5685-08.2009 19211884 PMC3904448

[B99] Zimmer T. S. Broekaart D. W. M. Gruber V. E. van Vliet E. A. Mühlebner A. Aronica E. (2020). Tuberous sclerosis complex as disease model for investigating mTOR-related gliopathy during epileptogenesis. *Front. Neurol.* 11:1028. 10.3389/fneur.2020.01028 33041976 PMC7527496

